# Targeting EHMT2 enhances NK cell maturation and antitumor activity

**DOI:** 10.1038/s41419-026-09124-y

**Published:** 2026-07-24

**Authors:** Minghang Yu, Xinhua Liu, Wenxiu Zhang, Xuefeng Huang, Yue Wu, Xinying Wang, Yuanyuan Chen, Ning Ding, Zilong Zhao, Xi Wang, Yang Li

**Affiliations:** 1 National Key Laboratory of Intelligent Tracking and Forecasting for Infectious Diseases, Beijing Key Laboratory of Viral Infectious Diseases, Institute of Infectious Diseases, National Center for Infectious Diseases, Beijing Ditan Hospital, Capital Medical University, Beijing, China; 2 Beijing Institute of Infectious Diseases, Beijing, China; 3 Department of Oncology, Capital Medical University, Beijing, China; 4 Department of Biochemistry and Molecular Biology, School of Basic Medicine Sciences, Hangzhou Normal University, Hangzhou, China; 5 Department of Gynecology and Obstetrics, Tianjin Key Laboratory of Female Reproductive Health and Eugenics, Tianjin Medical University General Hospital, Tianjin, China; 6 Key Laboratory of Carcinogenesis and Translational Research, Laboratory of Lymphoma Translational Research, Peking University Cancer Hospital & Institute, Beijing, China; 7 Department of Neurosurgery, Tianjin Institute of Neurology, Tianjin Medical University General Hospital, Tianjin, China; 8 Precision Medicine Center, Tianjin Medical University General Hospital, Tianjin, China

**Keywords:** NK cells, Differentiation, Immunotherapy

## Abstract

Natural killer (NK) cells are critical for both innate and adaptive immunity, and their effective regulation holds significant promise for tumor immunotherapy. The terminal maturation of NK cells is closely associated with enhanced effector functions, but the role of epigenetic mechanisms in this process remains incompletely understood. Here, we identify the histone methyltransferase EHMT2 as an epigenetic regulator that restrains NK cell maturation and function. EHMT2 expression is markedly downregulated upon cytokine activation in human NK cells and inversely correlated with effector gene expression across tissues and cancer types. NK cell-specific deletion of Ehmt2 in mice promotes terminal NK cell maturation, augmenting IFN-γ production and cytotoxicity both in vitro and in vivo. Integrated RNA-seq and H3K9me2 CUT&Tag profiling reveal that Ehmt2 loss reduces the repressive histone mark H3K9me2 and derepresses transcriptional programs associated with NK cell differentiation and cytotoxicity. Pharmacological inhibition of EHMT2 recapitulated these effects in both human umbilical cord blood (hUCB)-hematopoietic stem cell (HSC)-derived NK cells and peripheral blood NK cells, promoting terminal maturation and tumor cell killing. These findings define EHMT2-dependent H3K9me2 deposition as a key epigenetic barrier to NK cell effector programming and suggest that targeting EHMT2 may potentiate NK cell-based immunotherapies.

**Figure:**
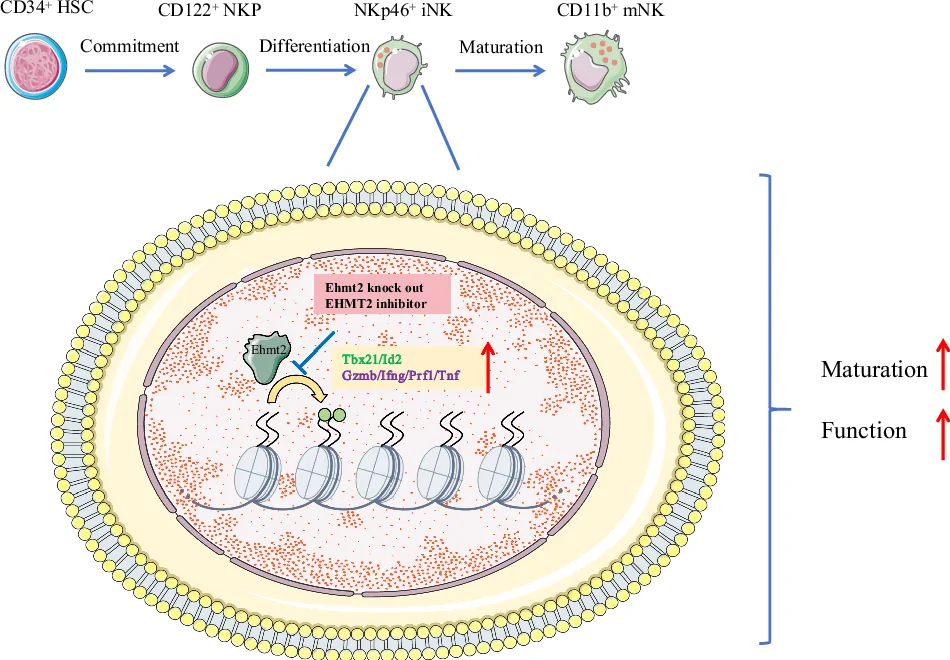

## Introduction

Natural killer (NK) cells are pivotal innate effectors in host defense against malignancies. Compared to T cell-based therapies, NK cell approaches offer distinct advantages, including “off-the-shelf” availability and superior safety profiles [1]. However, their clinical translation is currently hindered by limited persistence, insufficient tumor infiltration, and functional suppression within the tumor microenvironment [1–3]. NK cell effector function is intrinsically coupled with terminal maturation [4–6]. Clinically, a high frequency of terminally mature NK cells correlates with reduced disease burden and improved patient survival [7]. Therefore, strategies that promote NK cell maturation are urgently needed to optimize therapeutic outcomes. While the transcriptional network governing NK development is well-charted, the epigenetic checkpoints that constrain this process remain a critical knowledge gap. Through transcriptomic profiling of activated NK cells, we identified a marked downregulation of Euchromatic Histone Lysine Methyltransferase 2 (EHMT2), suggesting it may function as a barrier to activation. EHMT2 is the primary enzyme responsible for depositing repressive H3K9me2 marks, yet its specific role in NK cell biology remains undefined.

Here, we employed conditional Ehmt2 knockout mice and pharmacological inhibition to elucidate the epigenetic regulation of NK cell function. We demonstrate that EHMT2 acts as an intrinsic brake on NK cell development; its ablation reduces H3K9me2 deposition, thereby unleashing the transcriptional program required for terminal maturation and anti-tumor cytotoxicity. These findings highlight EHMT2 targeting as a promising strategy to potentiate NK cell-based immunotherapies.

## Results

### EHMT2 expression negatively correlates with NK cell effector function

To investigate epigenetic regulation during NK cell activation, we re-analyzed the public RNA-seq dataset GSE22919, which includes NK cells stimulated with IL-2, IL-12, and IL-18 (Fig. S1A, B). RNA-seq analysis confirmed robust reprogramming toward a cytotoxic state, with DEGs significantly enriched in lymphocyte activation and cytotoxicity pathways (Fig. S1C, D). Notably, among epigenetic modifiers, EHMT2 exhibited the most profound downregulation upon activation, a finding validated by RT-qPCR (Fig. S1E, F). Given EHMT2’s role as a repressive H3K9me2 writer, its downregulation suggests a mechanism to relieve epigenetic constraints on immune function genes.

**Fig. 1:**
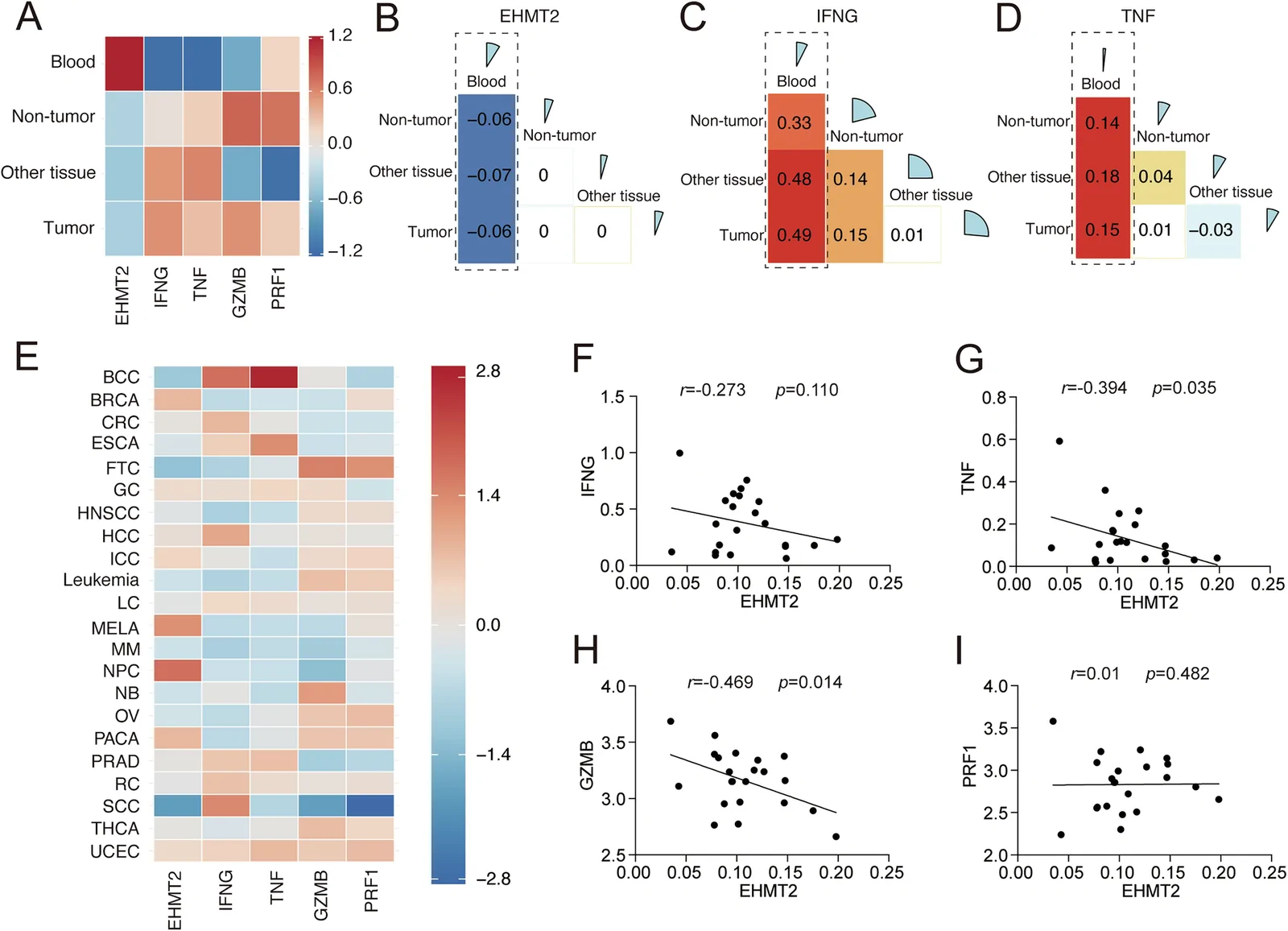
EHMT2 expression negatively correlates with NK cell effector function. **A** The heatmap illustrates the distribution of median expression levels for specified genes across different tissue groups, standardized using Z-scores. The color scheme reflects Z-score normalization, with expression values ranging from -1.2 (blue) to +1.2 (red). **B**–**D** Heatmaps depict expression variations of EHMT2, IFNG, and TNF in tumor, non-tumor, blood, and other tissue categories. Each box represents pairwise comparison test results between the group indicated in the row header and the column header. Numerical values within boxes denote log_2_FoldChange, while color saturation corresponds to differential significance (darker hues indicate higher significance; default significance threshold by 0.05 and only the significant comparisons are marked). Red/blue coloring exclusively reflects positive/negative log_2_FoldChange values. Pie charts display the percentage of cells expressing each gene within respective groups. **E** The heatmap illustrates the distribution of median expression levels for specified genes across different cancer types, standardized using Z-scores. The color scheme reflects Z-score normalization, with expression values ranging from -2.8 (blue) to +2.8 (red). **F**–**I** One-tailed Pearson correlation analysis between the expression of EHMT2 and the expression of IFNG, TNF, GZMB and PRF1 in different cancer types (each dot represents a cancer type; *n* = 22). The analysis was performed using the scDVA tool.

To further explore the relationship between EHMT2 and NK cell functionality, the single-cell RNA-seq data visualization and analysis (scDVA) tool was utilized [8]. This database integrates 22 cancer types, comprising 1223 samples from 716 patients (including tumor tissues, adjacent non-tumor tissues, peripheral blood, and other tissues such as lymph nodes), alongside 60 healthy controls. First, we analyzed EHMT2 and NK cell effector genes (IFNG, TNF, GZMB and PRF1) in CD56^dim^CD16^hi^ NK cells across tumor, non-tumor, blood, and other tissues (Figs. 1A–D and S2A, B). EHMT2 expression was significantly downregulated in tumor, non-tumor, and other tissues compared to blood (Fig. 1B). In contrast, effector genes IFNG and TNF exhibited markedly elevated expression in tumor, non-tumor, and other tissues relative to blood (Fig. 1C, D). The expression of GZMB and PRF1 is elevated in non-tumor and tumor tissues compared to blood, although the increase in PRF1 within tumors is not significant (Fig. S2A, B). Next, we examined EHMT2 and NK cell effector genes (IFNG, TNF, GZMB and PRF1) expression across distinct cancer types (Fig. 1E–I). Using Pearson correlation analysis, we assessed the associations between EHMT2 and each effector gene (IFNG, TNF, GZMB and PRF1) in malignancies. EHMT2 exhibited negative correlations with IFNG, TNF and GZMB expression (Fig. 1F–H), with statistically significant inverse relationships observed for TNF and GZMB (*p* < 0.05, Fig. 1G, H). In summary, these correlative results suggest that the expression level of EHMT2 is inversely associated with the effector genes expression of NK cells, providing a rationale to hypothesize that it may act as an epigenetic barrier to NK cell activation.

### Loss of Ehmt2 promotes terminal NK cell maturation

**Fig. 2:**
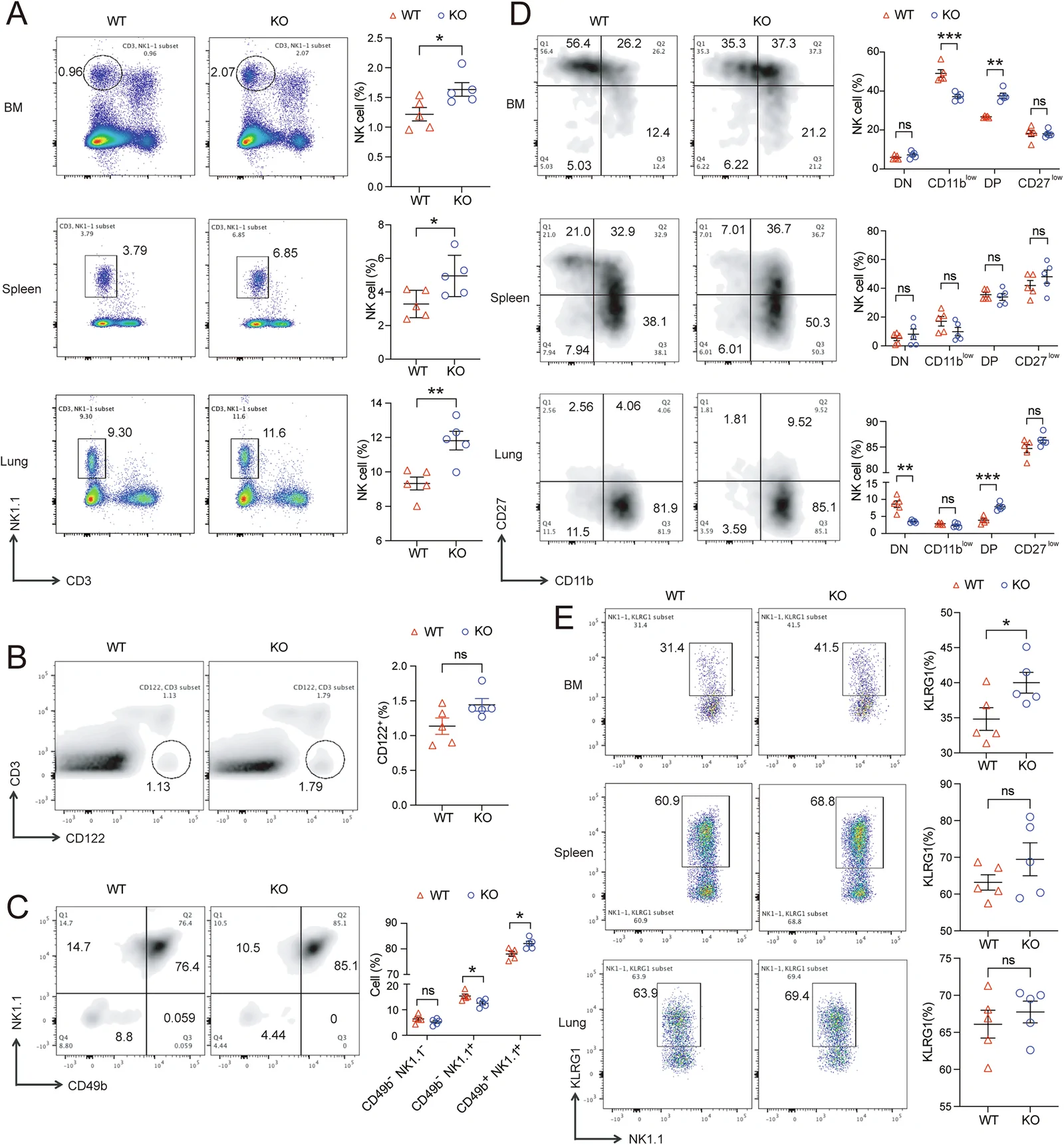
Loss of Ehmt2 promotes NK cell terminal maturation. **A** Flow cytometry plots (left) and scatter plots (right) show the percentage of NK cells (CD3^−^NK1.1^+^) in the BM, spleen and lung from WT and KO mice. **B** Flow cytometry plots (left) and scatter plots (right) show the percentage of NK cells (CD3^−^CD122^+^) in the BM. **C** Flow cytometry plots (left) and scatter plots (right) show the percentages of NKp (NK1.1^−^CD49b^−^), imNK (NK1.1^+^CD49b^−^), and mNK (NK1.1^+^CD49b^+^) cells gated from CD3^−^CD122^+^ splenocytes from WT and KO mice. **D** Flow cytometry plots (left) and scatter plots (right) show the percentage of NK cell subsets gated from CD3^−^NK1.1^+^ cells in BM, spleen, and lung based on CD27 and CD11b expression. **E** Flow cytometry plots (left) and scatter plots (right) show KLRG1 expression in the BM, spleen and lung CD3^−^NK1.1^+^ NK cells from WT and KO mice. The data are shown as the mean ± SEM (*n* = 5). *p < 0.05; **p < 0.01; ***p < 0.001.

Given that NK cell effector capability is intrinsically tied to terminal maturation, we hypothesized that the inverse correlation between EHMT2 and NK cell activation observed in clinical datasets might stem from a terminal maturation blockade. To investigate this systematically, Ehmt2 conditional knockout (hereafter referred to as KO) mice were generated by crossing Ehmt2^flox/flox^ mice (Fig. S3A, B) with Ncr1^*iCre*^ mice. Complete deletion of Ehmt2 in splenic CD3^−^NK1.1^+^ NK cells from KO mice was confirmed by Western blot analysis (Fig. S3C). Subsequent analysis revealed a significant reduction of H3K9me2, a histone modification catalyzed by Ehmt2, in KO NK cells (Fig. S3C). Compared with Ehmt2^flox/flox^ (hereafter referred to as WT) mice, KO mice exhibited significantly increased proportions of NK cells (CD3^−^NK1.1^+^) in BM, spleen and lung tissues (Fig. 2A). Although KO mice exhibited a trend toward increased CD3^−^CD122^+^ NK progenitor cells in BM, the difference was not statistically significant (Fig. 2B). Notably, Ehmt2 deficiency significantly altered the proportion of NK maturation subsets: mature NK cells (mNK; CD122^+^NK1.1^+^CD49b^+^) were markedly increased, whereas immature NK cells (imNK; CD122^+^NK1.1^+^CD49b^−^) showed substantial reduction (Fig. 2C). Furthermore, KO mice demonstrated significantly elevated frequencies of double-positive (DP; CD27^+^CD11b^+^) NK cells in BM and lung tissues, accompanied by decreased frequencies of CD11b^low^ (CD27^+^CD11b^-^) subsets in BM and double-negative (DN; CD27^−^CD11b^−^) subsets in lung tissue (Fig. 2D). KLRG1 marker analysis revealed significantly higher frequencies of terminally mature NK cells (CD3^−^NK1.1^+^KLRG1^+^) in KO BM compared to WT controls, though no significant differences were observed in spleen or lung tissues (Fig. 2E). These findings collectively indicate that Ehmt2 negatively regulates the terminal maturation of NK cells.

### Ehmt2 deficiency promotes NK cell antitumor immunity

**Fig. 3:**
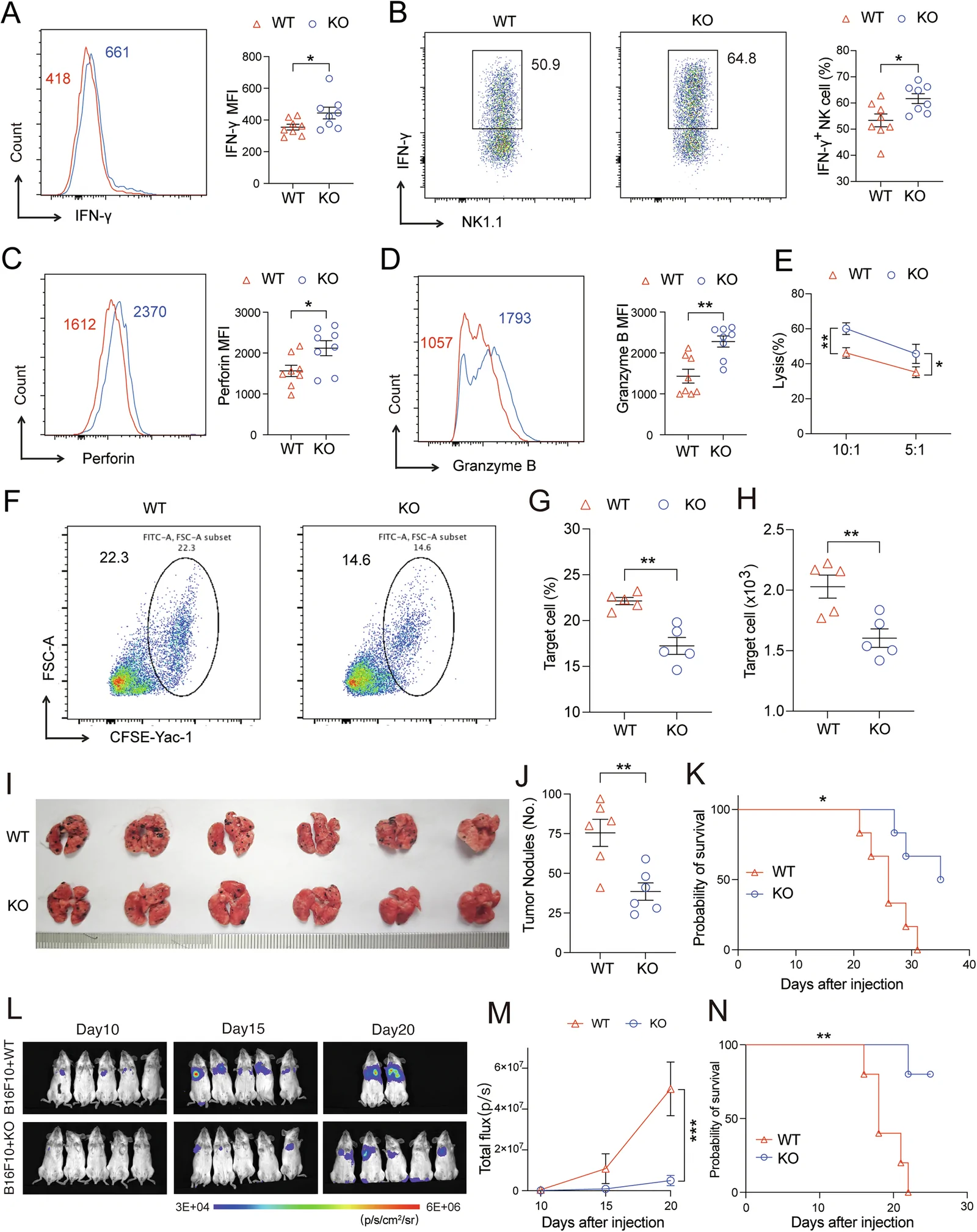
Ehmt2 deficiency promotes NK cell antitumor immunity. **A** Flow cytometry plot (left) and scatter plot (right) show intracellular staining of IFN-γ by splenic NK cells in WT or KO mice (*n* = 8). **B** Flow cytometry plots (left) and scatter plot (right) show the proportion of IFN-γ producing NK cells in WT or KO mice (*n* = 8). The cells were co-stimulated with PMA and ionomycin in the presence of GolgiStop. **C**, **D** Flow cytometry plots (left) and scatter plots (right) show intracellular staining of Perforin and Granzyme B by splenic NK cells in WT or KO mice (*n* = 8). **E** Yac-1 cells specifically lysed by splenic NK cells from WT or KO mice at the indicated E:T ratios (*n* = 3 per group; at least two independent experiments). **F** WT and KO mice were injected intraperitoneally with CFSE-labeled Yac-1 cells. The frequency (**G**) and number (**H**) of remaining CFSE-labeled Yac-1 cells in the peritoneal cavity were assessed 24 h post-injection. In vivo pulmonary metastasis model. Representative images of lung surfaces (**I**) and quantification of lung tumor nodules (**J**) from WT and Ehmt2 KO mice (*n* = 6) at 15 days post-intravenous (*i.v*.) injection with 2 × 10^5^ B16F10 cells. **K** Kaplan–Meier curves demonstrating the survival probability of the experimental mice. **L**–**N** In vivo adoptive transfer model. 1 × 10^6^ IL-15-expanded NK cells from WT or KO mice were injected *i.v*. into NSIG mice (*n* = 5). One day later, the mice were challenged with an *i.v*. injection of 1 × 10^5^ luciferase-tagged B16F10 cells. **L** Representative whole-body bioluminescent images showing the progression of tumor burden at days 10, 15, and 20 post-tumor injection. **M** Quantification of the bioluminescence signal (total flux) for mice receiving WT or KO NK cells over the 20-day monitoring period. Five mice per group were analyzed at days 10 and 15; at day 20, two surviving mice in the WT NK cell transfer group and five mice in the KO NK cell transfer group were analyzed. **N** Kaplan–Meier curves demonstrating the survival of the treated NSIG mice. The data are shown as the mean ± SEM. *p < 0.05; **p < 0.01; ***p < 0.001.

NK cell maturation is closely associated with enhanced effector function. To comprehensively evaluate the antitumor effects of Ehmt2-deficient NK cells, we conducted a series of analyzes both in vitro and in vivo*.* As the sole member of type II interferon, IFN-γ regulates antitumor immune responses through multiple pathways. Intracellular staining analysis showed that, compared with WT mice, NK cells from KO mice exhibited a significantly increased mean fluorescence intensity (MFI) of IFN-γ under resting conditions (Fig. 3A). Moreover, upon activation, the proportion of IFN-γ-producing NK cells was also markedly elevated (Fig. 3B). The cytotoxic function of NK cells relies on effector molecules within cytotoxic granules, such as Perforin and Granzyme B. Flow cytometry analysis revealed increased expression levels of Perforin and Granzyme B (Fig. 3C, D). Furthermore, we performed RT-qPCR assays, which confirmed significant upregulation of Ifng, Prf1 and Gzmb mRNA levels (Figs. S3D, E and 5I). To further investigate the inhibitory role of Ehmt2 in NK cell cytotoxicity, we compared the lytic activity of splenic NK cells from WT and KO mice against murine lymphoma Yac-1 cells. In cytotoxicity assays across varying effector-to-target (E:T) ratios, Ehmt2-deficient NK cells exhibited significantly enhanced killing activity against Yac-1 cells compared to WT control (Fig. 3E). To assess the in vivo tumor clearance capacity of Ehmt2-deficient NK cells, CFSE-labeled Yac-1 cells were intraperitoneally injected into WT and KO mice. Notably, KO mice displayed a substantial reduction in residual Yac-1 cell numbers (Fig. 3F–H). Furthermore, we challenged WT and KO mice with B16F10 cells, followed by the analysis of pulmonary metastatic growth. WT mice exhibited significantly more severe lung metastasis compared to KO mice (Fig. 3I), as determined by counting the number of lung tumor nodules (Fig. 3J). Consequently, KO mice exerted a stronger antitumor effect and significantly prolonged survival (Fig. 3K). To confirm this suppression of tumor progression is intrinsic to NK cells, we adoptively transferred an equal number of NK cells from WT or KO mice into NSIG mice (which lack T, B, and NK cells), 1 day prior to an injection of luciferase-tagged B16F10 tumor cells. We observed a significantly higher tumor burden in mice transferred with WT NK cells compared to KO NK cells, as determined by whole-body bioluminescent imaging (Fig. 3L, M). Furthermore, mice injected with KO NK cells exhibited prolonged survival (Fig. 3N). These data indicate that Ehmt2 deficiency significantly enhances NK cell effector functions and antitumor activity.

### Integrative RNA-seq and H3K9me2 CUT&Tag analysis reveals Ehmt2 as a key epigenetic repressor of NK cell differentiation and cytotoxicity

**Fig. 4:**
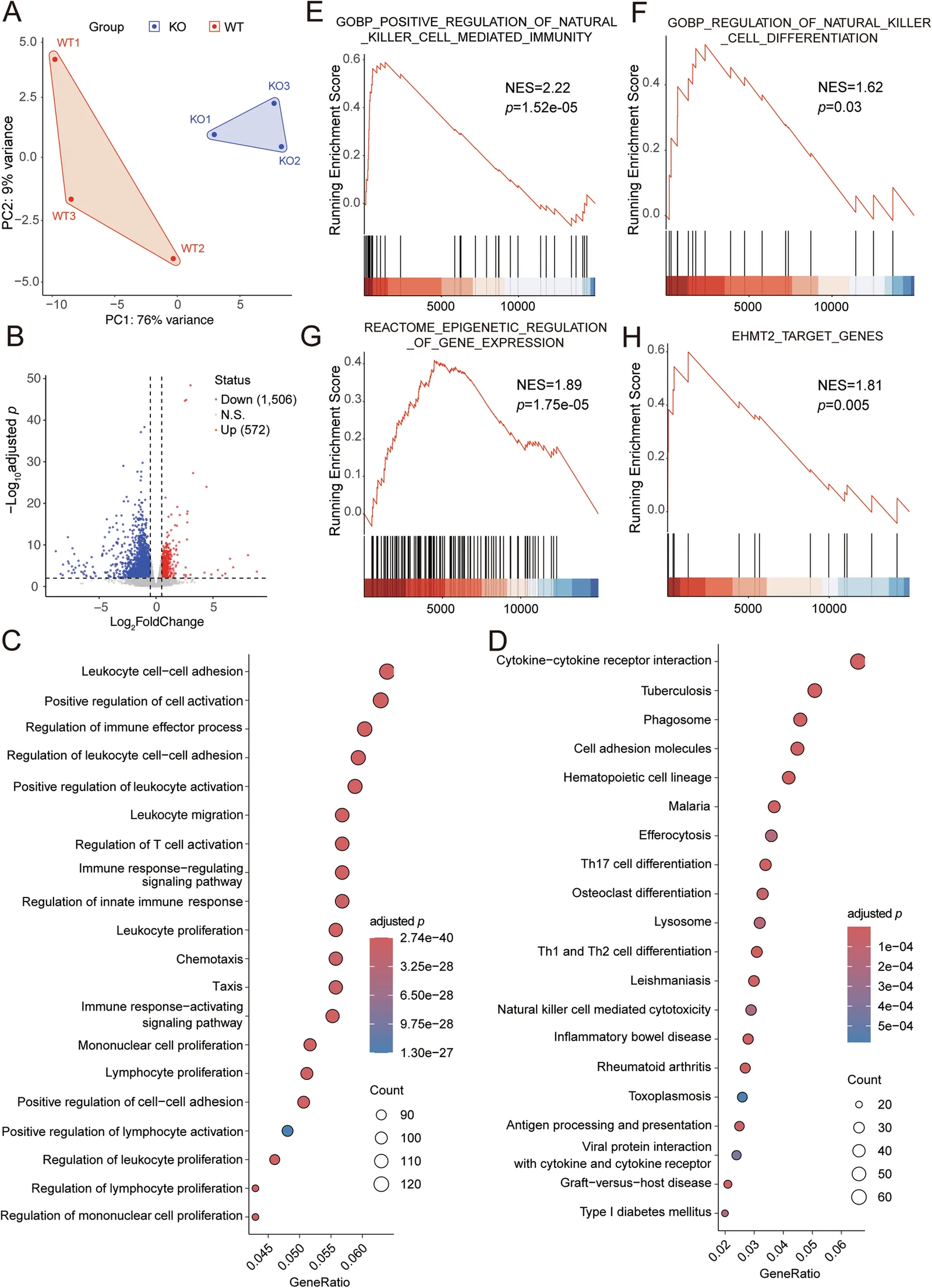
RNA-seq analysis reveals Ehmt2 deficiency promotes the differentiation of NK cells with enhanced effector function. **A** PCA analysis reveals transcriptional differences in splenic NK cells from WT or KO mice. **B** Volcano plots show the DEGs screened by |log_2_FoldChange| > 0.5 and adjusted *p* < 0.01 in RNA-seq. Red and blue dots represent upregulated and downregulated genes, respectively. GO **C** and KEGG **D** pathway enrichment results of DEGs in KO versus WT NK cells. Dot sizes represent the counts of DEGs in a pathway, and dot colors from red to blue indicate adjusted *p* values from low to high. GSEA illustrating enrichment of regulation of natural killer cell mediated immunity (**E**), regulation of natural killer cell differentiation (**F**), epigenetic regulation of gene expression (**G**) and EHMT2 target genes (**H**) gene sets in KO versus WT NK cells.

To determine whether Ehmt2 deletion induces broader alterations in NK cell state, we performed transcriptomic profiling of sorted splenic NK cells from WT and KO mice. Principal component analysis (PCA) revealed a clear separation between WT and KO NK cells, indicating a global shift in transcriptional identity (Fig. 4A). In summary, a total of 1506 genes were found to be downregulated, and 572 genes were found to be upregulated in KO NK cells in comparison with WT NK cells (|log_2_FoldChange| > 0.5 and adjusted *p* < 0.01, Fig. 4B). GO analysis identified pronounced enrichment in biological processes associated with positive regulation of cell activation, leukocyte proliferation, and leukocyte migration (Fig. 4C). KEGG pathway analysis revealed significant enrichment of differentially expressed genes in pathways associated with cell differentiation (e.g., Th1 and Th2 cell differentiation and Th17 cell differentiation) and natural killer cell mediated cytotoxicity (Fig. 4D). Notably, Gene Set Enrichment Analysis (GSEA) demonstrated that Ehmt2 deficiency positively correlated with gene sets involved in regulation of natural killer cell mediated immunity, regulation of natural killer cell differentiation, Ehmt2 target genes and epigenetic regulation of gene expression (Fig. 4E–H). The GSEA results demonstrate that genetic ablation of Ehmt2 leads to the coordinated upregulation of gene programs critical for NK cell biology, including differentiation and activation. The enrichment of epigenetic regulatory pathways, together with the enrichment of known Ehmt2 target genes, supports a role for Ehmt2 in shaping the NK cell transcriptional landscape. We further visualized the expression of NK activity-associated genes in different categories, including activating receptors, inhibitory receptors, transcription factors, effector genes and cytokine receptors. Compared with WT NK cells, most activating receptors, inhibitory receptors, and cytokine receptors showed an upward trend in expression in KO NK cells, while the expression of key transcription factors and effector genes increased (Fig. S4A–E). These data indicate that Ehmt2 deficiency reshapes NK cell transcriptional programs at a systems level, promoting a gene expression landscape associated with enhanced functional competence.

**Fig. 5:**
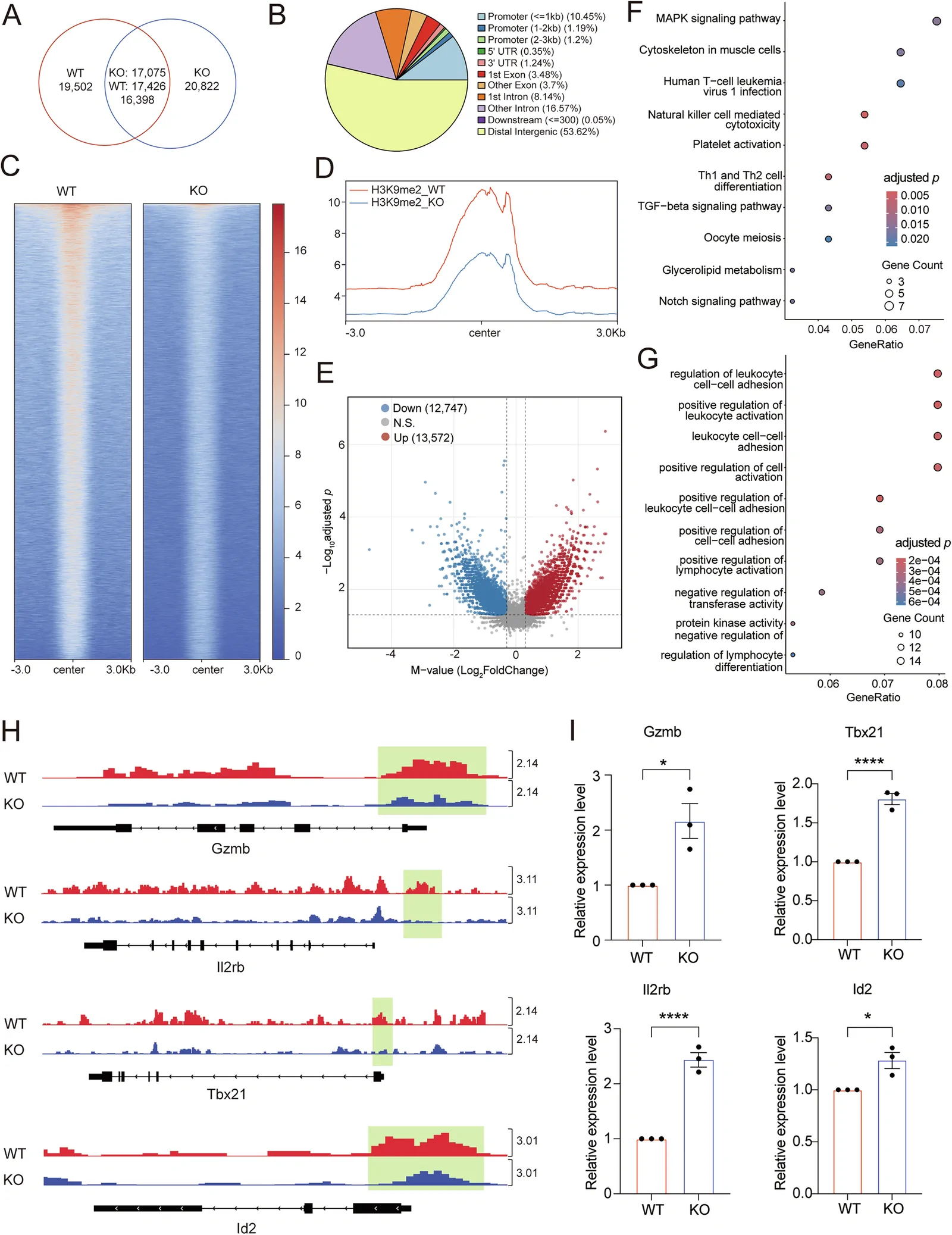
CUT&Tag analysis shows genome-wide influence of Ehmt2 knockout on H3K9me2 deposition and its functional consequences. **A** Overlap of H3K9me2 peaks between WT and KO NK cells. **B** Genomic annotation of the overlapping H3K9me2 peaks. **C** H3K9me2 signal intensity at merged peaks in WT and KO NK cells. **D** Density profile of H3K9me2 signal across merged peaks in WT and KO NK cells. **E** Volcano plot illustrating the differential peaks between WT and KO NK cells. The significantly differential peaks were identified by the thresholds of |log_2_FoldChange| > 0.3 and adjusted *p* < 0.05. **F** The top 10 significantly enriched KEGG pathways. **G** The top 10 significantly enriched GO biological processes. Dot sizes represent number of genes in a pathway, and dot colors from red to blue indicate adjusted *p* values from low to high. **H** H3K9me2 signal profiles at selected NK cell effector genes in WT and KO NK cells. **I** Gzmb, Il2rb, Tbx21 and Id2 mRNA expression detected by RT-qPCR in WT and KO NK cells. The data are shown as the mean ± SEM. *p < 0.05; ****p < 0.0001.

To investigate the epigenetic basis of this transcriptional reprogramming, we performed H3K9me2 CUT&Tag profiling. We identified 36,928 and 37,897 high-confidence H3K9me2 peaks in WT and KO samples, respectively, among which 16,398 common regions were identified (Fig. 5A). Genomic annotation showed that H3K9me2 peaks were predominantly located in distal intergenic and intronic regions, while a small fraction mapped to promoter regions, consistent with H3K9me2 being a repressive mark largely enriched in heterochromatic regions (Fig. 5B). Heatmap and density profile plots demonstrated a global reduction of H3K9me2 signal intensity in KO NK cells compared with WT NK cells, confirming efficient depletion of this repressive histone modification following Ehmt2 loss (Fig. 5C, D). To further investigate the impact of Ehmt2 knockout on H3K9me2 signals, we performed differential peak analysis using MAnorm. Using a threshold of |log_2_FoldChange| > 0.3 and adjusted *p* < 0.05, we identified a total of 12,747 downregulated and 13,572 upregulated peaks in KO NK cells compared to WT NK cells (Fig. 5E). To functionally link the loss of H3K9me2 to transcriptional changes, we intersected genes that were both upregulated in KO and exhibited loss of H3K9me2 at their loci, which obtained a total of 199 genes (Fig. S4F). The intersected gene set was significantly enriched in lymphocyte differentiation and cytotoxicity related pathways, including Natural killer cell mediated cytotoxicity, Th1 and Th2 cell differentiation, and MAPK signaling pathway (Fig. 5F). GO enrichment further highlighted functions such as positive regulation of lymphocyte activation, leukocyte cell-cell adhesion, and regulation of lymphocyte differentiation (Fig. 5G), indicating that Ehmt2-dependent H3K9me2 plays a suppressive role in NK cell activation and effector differentiation. Consistent with this, we observed a pronounced reduction of H3K9me2 signal at key NK cell cytotoxicity gene Gzmb, receptor gene Il2rb, and transcription factor Tbx21 and Id2 (Fig. 5H). Finally, RT-qPCR analysis confirmed that the loss of H3K9me2 at these loci was associated with their transcriptional upregulation in KO NK cells (Fig. 5I). Collectively, these findings demonstrate that Ehmt2-mediated H3K9me2 deposition acts as a global epigenetic constraint on NK cell gene programs, and its loss results in coordinated derepression of functionally relevant transcriptional networks.

### Pharmacologic EHMT2 inhibition promotes human NK cell terminal maturation

**Fig. 6:**
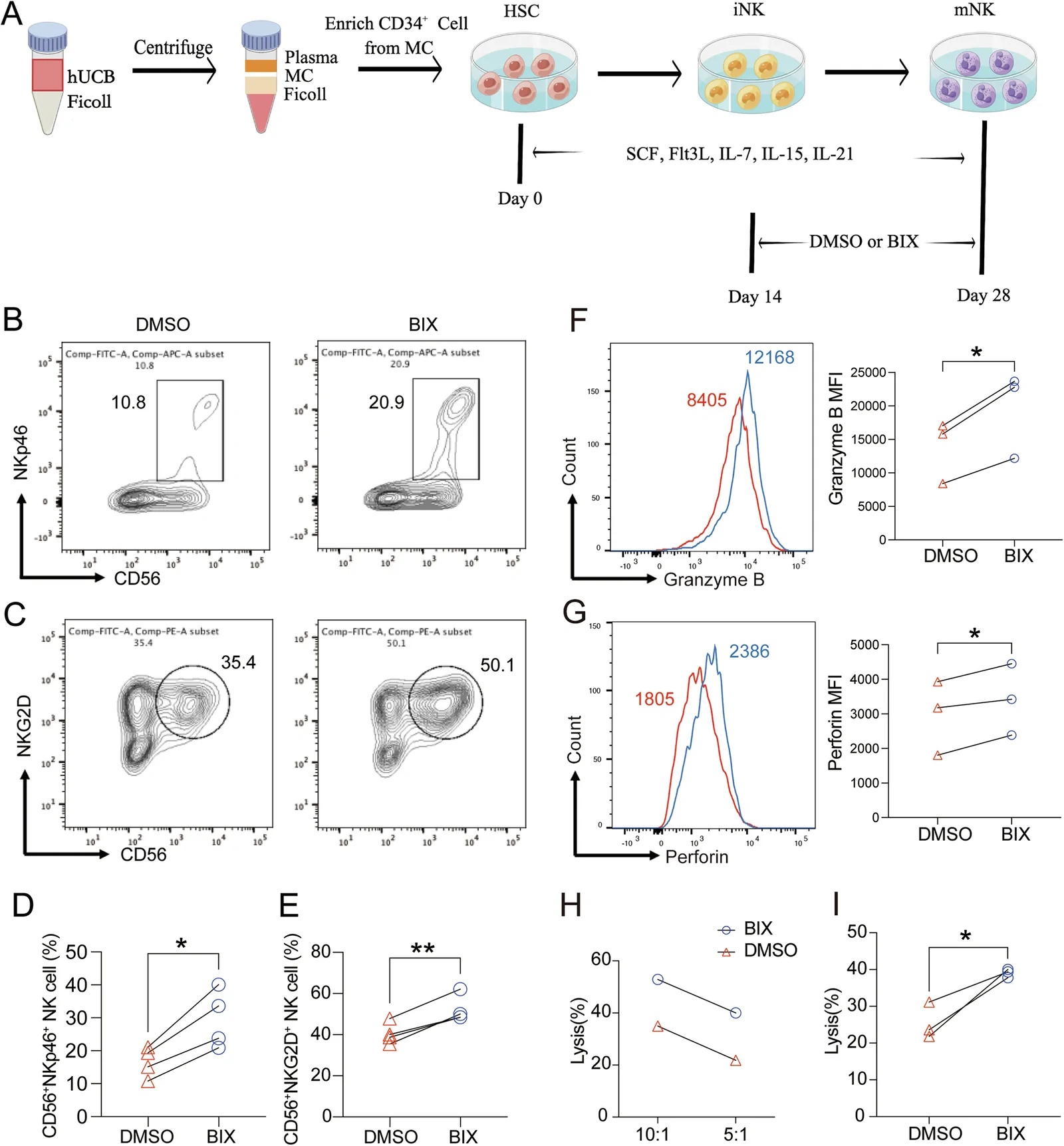
Inhibition of EHMT2 activity promotes human NK cell terminal maturation. **A** Schematic of in vitro human NK cell differentiation from hUCB-HSCs using a two-step culture system, with the addition of 0.25 μM BIX-01294 or DMSO during the process. Flow cytometry plots (**B**, **C**) and scatter plots (**D**, **E**) show the percentage of CD56^+^NKp46^+^ NK cells and CD56^+^NKG2D^+^ NK cells differentiated from hUCB-HSCs and treated with BIX-01294 or DMSO (*n* = 4 donors). **F**, **G** Flow cytometry plots (left) and scatter plots (right) show intracellular staining of Granzyme B and Perforin by NK cells in the BIX-01294 or DMSO groups (*n* = 3 donors). **H** Cell-mediated cytotoxicity of sorted NKG2D^+^CD56^+^ NK cells in the BIX-01294 or DMSO groups against K562 cell lines. Graphs show percent-specific lysis for NK cells from one representative donor. **I** Relative change in specific lysis was calculated for sorted NKG2D^+^CD56^+^ NK cells in the BIX-01294 or DMSO groups at a 5:1 E:T ratio (*n* = 3 donors). *p < 0.05; **p < 0.01.

To further investigate the impact of EHMT2 methyltransferase activity on human NK cell terminal development, we treated human umbilical cord blood (hUCB)-hematopoietic stem cells (HSCs) with small-molecule inhibitor BIX-01294 starting on day 14 of differentiation (Fig. 6A). Analysis of differentiation markers on day 28 revealed that inhibition of EHMT2 significantly enhanced NK cell maturation. Specifically, the percentages of CD56^+^NKp46^+^ and CD56^+^NKG2D^+^ cell populations were substantially higher in the BIX-01294-treated group than DMSO controls at day 28 (Fig. 6B–E). Furthermore, flow cytometry analysis demonstrated that the MFI of key cytotoxic granules, including both Granzyme B and Perforin, was significantly upregulated in the BIX-01294-treated group (Fig. 6F, G). The elevated expression of effector molecules directly translated into superior effector function, as evidenced by the enhanced cytotoxic capacity of sorted NKG2D^+^CD56^+^ NK cells against K562 target cells (Fig. 6H, I). Therefore, we conclude that EHMT2 plays a pivotal role in orchestrating the terminal maturation and conferring enhanced per-cell cytotoxic function.

### Inhibition of EHMT2 methyltransferase activity enhances human NK cell effector function

**Fig. 7:**
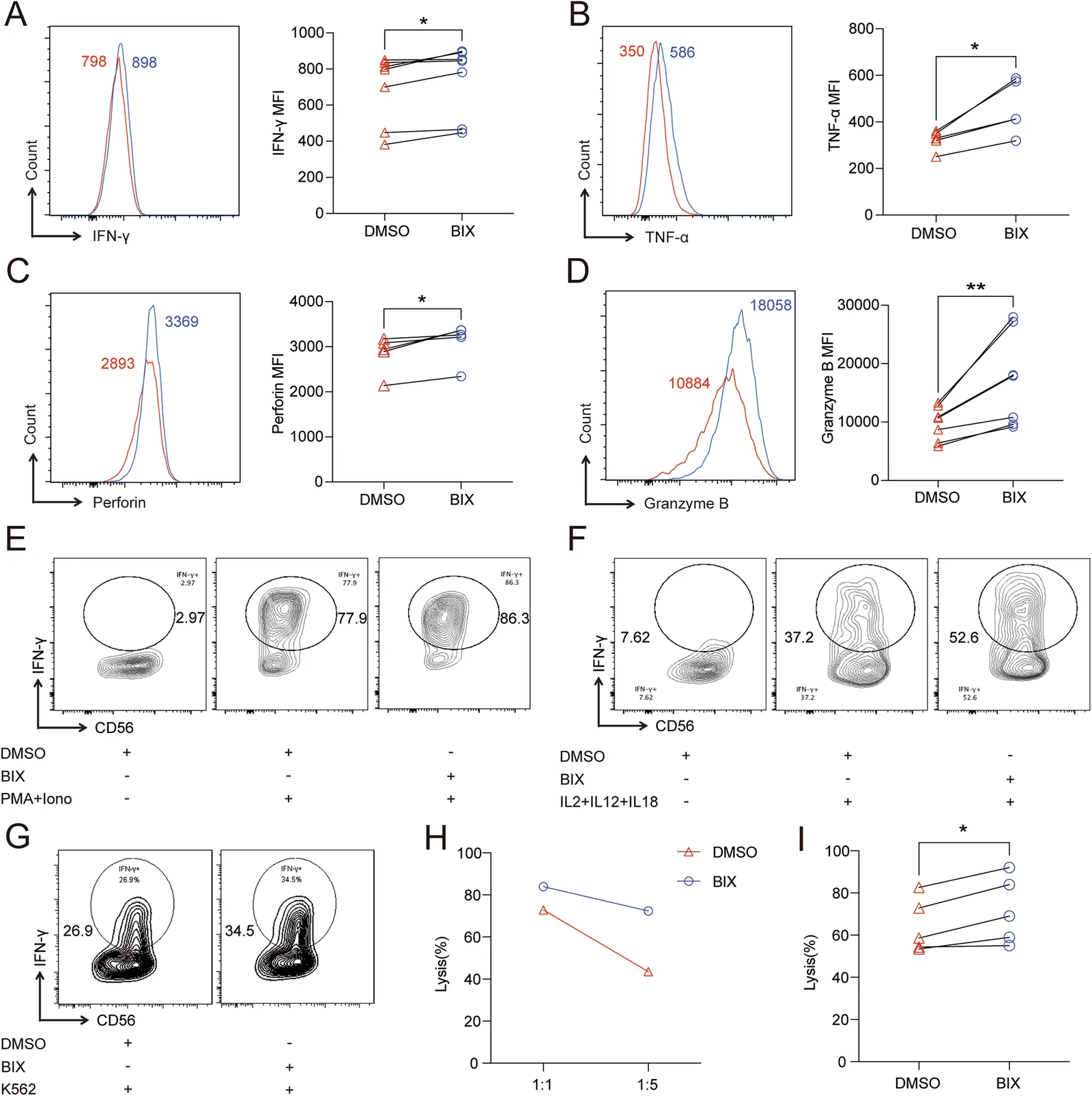
Inhibition of EHMT2 methyltransferase activity enhances NK cell effector functions and tumor-killing capacity. **A**–**D** Flow cytometry plots (left) and scatter plots (right) show intracellular staining of IFN-γ, TNF-α, Perforin and Granzyme B by unstimulated NK cells in the BIX-01294 or DMSO groups (*n* = 5-7 donors). **E**–**G** Flow cytometry plots show intracellular staining of IFN-γ production by NK cells in the BIX-01294 or DMSO groups. The cells were co-stimulated with PMA and ionomycin (**E**), IL-2 + IL-12 + IL-18 (**F**) or K562 cells (**G**) in the presence of GolgiStop. **H** Cell-mediated cytotoxicity of NK cells in the BIX-01294 or DMSO groups against HepG2 cell lines. Graphs show percent-specific lysis for NK cells from one representative donor. **I** Relative change in specific lysis was calculated for NK cells in the BIX-01294 or DMSO groups at a 1:1 E:T ratio (*n* = 5 donors). NK cells were isolated from donors and treated for 5 days with 0.25 μM BIX-01294 or DMSO. *p < 0.05; **p < 0.01.

To delineate the cell-intrinsic role of EHMT2 in regulating the function of human NK cells, we next examined the impact of the EHMT2 inhibitor BIX-01294 on peripheral blood NK cells. Initial Western blot analysis confirmed the efficacy of BIX-01294 treatment, showing a substantial reduction in global H3K9me2 levels (Fig. S5A). We first profiled the expression of key effector molecules in unstimulated NK cells. Flow cytometry analysis revealed that EHMT2 inhibition significantly altered the intracellular stores of IFN-γ, TNF-α, Perforin and Granzyme B (Fig. 7A–D), suggesting that EHMT2 inhibition modulates the baseline functional state of NK cells. We then assessed the functional competence of these cells under distinct activating conditions. Following stimulation with cytokines (IL-2 + IL-12 + IL-18), pharmacologic agents (PMA + ionomycin), or upon contact with K562 tumor cells, BIX-01294-treated NK cells exhibited a markedly enhanced capacity to produce IFN-γ compared to DMSO controls, as quantified by the percentage of positive cells (Fig. 7E–G). This potentiated cytokine response was further validated at the transcriptional level, where we observed a significant increase in *IFNG* mRNA expression in BIX-01294-treated cells after PMA + ionomycin activation (Fig. S5B). Critically, this augmented effector profile translated into superior cytotoxic function. In a cytotoxicity assay, NK cells pre-treated with BIX-01294 demonstrated significantly enhanced lytic activity against HepG2 target cells across multiple E:T ratios (Fig. 7H, I). Collectively, these data demonstrate that pharmacological inhibition of EHMT2 augments the cytotoxic and cytokine-producing functions of human NK cells, positioning EHMT2 as a key epigenetic checkpoint constraining their effector potential.

We further validated these observations in the human NK92MI cell line, where EHMT2 inhibition similarly recapitulated the enhanced effector phenotype (Fig. S5C–J). To elucidate the underlying molecular mechanisms, we performed RNA-seq on BIX-01294-treated NK92MI cells. PCA revealed a distinct transcriptomic shift, with 1433 differentially expressed genes (810 upregulated and 623 downregulated, |log2FoldChange| > 0.5 and adjusted *p* < 0.01) identified (Fig. S6A, B). KEGG and GO highlighted a significant activation of pathways governing NK cell cytotoxicity and lymphocyte differentiation (Fig. S6C, D). Consistently, GSEA confirmed the upregulation of gene sets driving positive regulation of NK cell activation (Fig. S6E, F). Heatmap analysis revealed a concerted upregulation of cytokine receptors and cytotoxic mediators upon EHMT2 inhibition, providing a clear transcriptional basis for the enhanced functional capacity (Fig. S6G, H). In contrast, activating and inhibitory receptors displayed heterogeneous expression patterns, suggesting a nuanced recalibration of target recognition mechanisms rather than global activation (Fig. S6I, J). Collectively, these data demonstrate that EHMT2 inhibition orchestrates broad transcriptional reprogramming, effectively shifting NK cells toward a more activated effector state.

To exclude the potential off-target effects of the EHMT2 inhibitor BIX-01294 and confirm that the observed functional enhancement was specifically mediated through EHMT2, we performed a series of genetic and pharmacological validation experiments. We first utilized BIX-01294-treated KO NK cells as a control. As shown in Fig. S7A–E, while BIX-01294 significantly enhanced the effector functions of WT NK cells, it failed to further increase the expression of IFN-γ, Perforin, Granzyme B, or overall cytolytic activity in KO NK cells. Next, we employed UNC0642, an alternative EHMT2 inhibitor, to treat human NK cells (Fig. S7F–K). Similar to the effects of BIX-01294, UNC0642 treatment significantly increased the MFI of IFN-γ, Perforin and Granzyme B (Fig. S7G–I) and enhanced the killing capacity of NK cells against target cells (Fig. S7J, K). Collectively, these results demonstrate that the immunomodulatory effects of these inhibitors are specifically driven by EHMT2 inhibition.

It is noted that the phenotypic changes (IFN-γ, TNF-α and Perforin) in BIX-01294-treated human NK cells were more modest than those in KO mice NK cells (Fig. 7A–C). To exclude the possibility of incomplete pharmacological inhibition, we performed CRISPR/Cas9-mediated EHMT2 knockout in human NK92MI cells. This genetic ablation yielded comparable moderate enhancements rather than a drastic phenotypic amplification (Fig. S7L–O). Together, these findings confirm that both approaches independently validate EHMT2 as a conserved epigenetic checkpoint constraining NK cell potential. The more pronounced mice phenotypes likely reflect inherent species differences.

## Discussion

The therapeutic potential of NK cells is currently constrained by their limited persistence and susceptibility to suppression within the tumor microenvironment [1–3]. Since terminal maturation is intrinsically linked to enhanced cytotoxicity and improved clinical outcomes [7], identifying the regulatory checkpoints governing this process is critical. Here, we identify the histone methyltransferase EHMT2 as a key epigenetic repressor that restricts NK cell terminal maturation and effector function. By establishing EHMT2-mediated H3K9 methylation as a fundamental constraint on NK cell biology, our findings highlight EHMT2 inhibition as a promising strategy to unleash the full therapeutic potential of NK cell-based immunotherapies.

NK cell maturation is a tightly regulated process. Our findings elucidate a critical role of EHMT2 in suppressing the terminal maturation of NK cells. Genetic ablation of Ehmt2 in mice resulted in a significant increase in mature NK subsets (mNK and CD27^+^CD11b^+^ populations) and terminally differentiated KLRG1^+^ NK cells in BM. In hUCB-HSCs-derived cultures, EHMT2 inhibition increased the frequencies of CD56^+^NKp46^+^ and CD56^+^NKG2D^+^ cells, phenotypically consistent with enhanced maturation. Mechanistically, RNA-seq and H3K9me2 CUT&Tag analyzes revealed that deletion of Ehmt2 markedly reduces H3K9me2 deposition at key NK cell-associated loci, which correlates with upregulation of genes involved in differentiation (e.g., Tbx21 and Id2). GSEA analysis further revealed that Ehmt2 KO NK cells showed transcriptional upregulation of pathways associated with regulation of NK cell differentiation, Ehmt2 target genes and epigenetic regulation of gene expression, further indicating that Ehmt2 epigenetic silencing restricts NK maturation. These observations align with prior studies demonstrating Ehmt2’s role in maintaining cellular identity in CD8^+^ T cells by repressing alternative lineage genes during proliferation [9]. Similarly, in T cell development, Ehmt2 enforces lineage fidelity by silencing helper T cell genes in cytotoxic T cells [10], highlighting its conserved function as an epigenetic checkpoint across immune cell types. Our work extends this paradigm to NK cells, positioning Ehmt2 as a gatekeeper of maturation through chromatin-mediated repression.

Beyond its role in NK cell maturation, our findings reveal that EHMT2 constrains NK cell effector functions by repressing transcriptional programs that encode cytotoxic mediators and proinflammatory cytokines. Analysis of the scDVA database revealed a negative correlation between EHMT2 expression and NK cell effector genes. Furthermore, Ehmt2 deficiency significantly augmented mice NK cell effector functions, including elevated IFN-γ production and cytotoxicity against tumor cells both in vitro and in vivo. Notably, pharmacological inhibition of EHMT2 with BIX-01294 recapitulated many of these effects in human hUCB-HSCs-derived NK cells and peripheral blood NK cells. In a related context, a study by Jing et al. reported that EHMT2 acts as a repressor of T cell fate, and its pharmacological inhibition promotes the differentiation of pluripotent stem cells into T cells with enhanced effector functions [11]. Although the target cell lineages (T cells vs. NK cells) and developmental contexts differ, both studies converge on an evolutionarily conserved core mechanism: EHMT2-mediated H3K9 methylation serves as a fundamental epigenetic constraint that limits the effector programs of lymphocytes, including both T cells and NK cells. This mechanistic convergence is of significant scientific importance, suggesting that targeting EHMT2 could represent a broad-spectrum strategy to unleash the functional potential of multiple lymphocyte subsets.

An important aspect of our findings is that multiple NK cell maturation and functional parameters associated with Ehmt2 deletion are moderate in magnitude. While such effect sizes may appear limited when evaluated using criteria commonly applied to lineage-defining transcription factors (TFs), they are consistent with the established conceptual framework of epigenetic regulation. TFs act as the primary, instructive drivers of cell-specific transcriptional programs, meaning their absence typically causes drastic, qualitative disruptions in cell fate and differentiation [12, 13]. In contrast, chromatin regulators (CRs) and their associated epigenetic marks generally do not function as hardwired, deterministic binary switches [14]. Instead, they navigate the epigenetic landscape by acting as quantitative modulators-akin to a “volume dial” or dynamic “epigenetic barrier”-that fine-tune the timing, magnitude, and plasticity of gene expression [15, 16]. Rather than independently dictating absolute cellular identities, CRs establish dynamic epigenetic barriers to coordinate future transcriptional responsiveness and induction kinetics [16]. Consequently, whereas knocking out key TFs abrogates essential cellular trajectories, the targeted loss of specific CR catalytic activities frequently manifests as quantitative or moderate shifts in developmental and functional phenotypes [14], reflecting their fundamental role in modulating environmental adaptability and physiological plasticity rather than strictly defining basic cell fates. This conceptual framework provides a mechanistic explanation for the moderate yet consistent phenotypic shifts observed in our study. Viewed in this context, our data support a model in which EHMT2 acts as an epigenetic brake that constrains NK cell maturation and effector programming. Importantly, as evidenced by our in vivo tumor models, releasing this epigenetic brake ultimately coordinates to produce a biologically profound enhancement of systemic antitumor immunity and overall survival.

The translational potential of EHMT2 inhibition in cancer immunotherapy is substantial. EHMT2 overexpression in tumors fosters an immunosuppressive microenvironment by silencing chemokines critical for lymphocyte infiltration [17] and repressing tumor antigens [18]. EHMT2 inhibition in melanoma models synergizes with anti-PD-1 therapy to overcome resistance [19], while in colorectal cancer, EHMT2-mediated suppression of galectin-7 dampens CD8^+^ T cell responses [20]. Our work extends this paradigm to NK cells, proposing that EHMT2 blockade could augment both adaptive and innate antitumor immunity. Our data demonstrate that Ehmt2 ablation enhances NK cell antitumor activity, as evidenced by the rapid and significant clearance of tumor cells in vivo. Notably, both hUCB-HSCs-derived NK cells and peripheral blood NK cells that were treated with BIX-01294 exhibited enhanced cytotoxicity, suggesting the clinical potential of this approach for ex vivo engineering of NK cell therapies. Given EHMT2’s dual role in tumorigenesis and immune evasion, its inhibition may serve as a multipronged strategy to reprogram malignant cells and reinvigorate antitumor immunity, converting immunologically “cold” tumors into “hot” targets [21].

In summary, EHMT2 emerges as a pivotal epigenetic modulator of NK cell biology, constraining maturation and effector functions. Targeting EHMT2 holds promise for enhancing NK cell-based immunotherapies and sensitizing tumors to immune checkpoint blockade. Future studies should address tissue-specific effects of EHMT2 inhibition and optimize combinatorial regimens to maximize clinical efficacy.

## Materials and methods

### Mice

Ncr1^*iCre*^ mice were maintained as previously described [22, 23]. Ehmt2^flox/flox^ mice were generated by Cyagen Biosciences. Ncr1^*iCre*^ transgenic mice were crossed with Ehmt2^flox/flox^ mice to generate Ehmt2^flox/flox^; Ncr1^*iCre*^ knockout (KO) mice. NOD-PrkdcscidIL2rgtm1 Mice (NSIG) were provided by Hfk Biosciences. All experimental and control mice (Ehmt2-related lines) were on a pure C57BL/6J background. Age- and sex-matched mice between 8 and 12 weeks of age were used for all experiments. All mice were maintained in a specific pathogen-free facility for use according to the guidelines for experimental animals at Capital Medical University. Mice were randomly allocated to experimental groups. The investigators were not blinded to group allocation during the experiment and outcome assessment. All animal procedures were approved by the Animal Ethics Committee of Capital Medical University (AEEI-2024-062).

### Cell lines and cell culture

NK92MI cells were cultured in α-MEM medium (Gibco) containing 12.5% fetal bovine serum (FBS) (Gibco), 12.5% horse serum (HS) (Gibco), 0.2 mM inositol (Sigma), 0.1 mM β-ME (Gibco), 0.02 mM folic acid (Sigma), and 1×Penicillin-Streptomycin (PS) (Gibco). K562 and Yac-1 cells were cultured in RPMI-1640 medium (Gibco) containing 10% FBS (Gibco) and 1×PS. HepG2 and B16F10 cells were maintained in high glucose DMEM medium (Gibco) containing 10% FBS (Gibco) and 1×PS. The NK92MI cell line was purchased from American Type Culture Collection. K562, Yac-1, B16F10, HepG2 cell lines were obtained from the Cell Bank of the Chinese Academy of Sciences. The identity of NK92MI and B16F10 was authenticated by short tandem repeat (STR) profiling, and all cell lines were verified to be mycoplasma-free.

### Isolation and stimulation of NK cells

In vitro human NK cell differentiation assay was performed as previously described [24]. Peripheral blood mononuclear cells were obtained from the peripheral blood of healthy human donors and isolated by Ficoll gradient centrifugation, and then primary NK cells were isolated and purified using a negative selection isolation kit (Miltenyi Biotec). The isolated NK cells were subsequently cultured in CTS NK-Xpander Medium (Gibco) following the manufacturer’s instructions.

### In vitro human NK cell differentiation assay

The isolation of leukocytes from umbilical cord blood was conducted by means of density gradient centrifugation. CD34^+^ cells were subsequently enriched via immunomagnetic separation with a human CD34 positive selection kit (Stemcell Technologies). The purified CD34^+^ cell population was maintained in GMP Stem Cell Growth Medium (CellGenix) containing 10% fetal bovine serum (FBS; Gibco), 1% Penicillin-Streptomycin antibiotic cocktail (Gibco), 25 μg/mL gentamicin (Gibco), and the following recombinant human cytokines (all from PeproTech): stem cell factor (SCF), FMS-like tyrosine kinase 3 ligand (Flt3L), interleukin-7 (IL-7), interleukin-15 (IL-15), and interleukin-21 (IL-21), each supplemented at 20 ng/mL. Next, CD56^+^NKG2D^+^CD3^-^ NK cells were purified using a fluorescence-activated cell sorting Aria cell sorter from BD Biosciences.

### Generation of CRISPR-Cas9 mediated gene knockout cell lines

SgRNA oligonucleotide sequences targeting EHMT2 were annealed and cloned into the U6-sgRNA-EF1a-spCas9-FLAG-CMV-EGFP-P2A-puro plasmid (Genechem). To generate stable sgEHMT2 cell lines, NK92MI cells were infected using lentivirus containing medium supplemented with 10 μg/mL polybrene, and the medium was changed 12 h later. At 48 h post infection, 2 μg/mL puromycin was used to select and maintain transduced cells. The EHMT2 knockout efficiency was tested using Western blot. The sgRNA sequence targeting EHMT2 is as follows: 5′-CGGGCCAAGATGTCAATGAC-3′.

### Metastatic melanoma model

Luciferase-tagged B16F10 cells were injected *i.v*. into WT and KO mice. 15 d after injection, the mice were euthanized for postmortem analysis. Metastatic nodules in the lung were analyzed macroscopically and counted. Purified WT and KO NK cells were injected *i.v*. into NSIG mice; 1 day after injection, luciferase-tagged B16F10 cells were injected *i.v*. into NSIG mice. Metastases in the lung were assessed by bioluminescent imaging analyzes using the Xenogen IVIS Imaging system.

### Tissue collection and cell isolation

Lung tissue was minced into small pieces and digested in DMEM containing type I collagenase (Sigma-Aldrich). Digested lung tissue samples were pelleted, resuspended in 40% Percoll (Solarbio), and carefully underlaid with 70% Percoll in a 15 ml tube. After centrifugation, leukocytes were enriched at the interface between the 40% and 70% Percoll layers. Spleens were collected in PBS and mashed with a syringe plunger. BM cells were harvested by flushing the tibiae and femurs with PBS. To obtain final single-cell suspensions, the processed lung, spleen, and BM cells were passed through 40 μm cell strainers.

### Flow cytometry

All antibodies were purchased from BioLegend, Thermo Fisher Scientific or BD Biosciences (Table S1). Standard protocols were followed for flow cytometry. Briefly, for analysis of surface markers, cells were stained in PBS containing 2% FBS at room temperature. Cytokine staining was performed using Fixation/Permeabilization Solution Kit (BD Biosciences) following the manufacturer’s instructions. Stained splenocyte samples were run on BD LSRFortessa using FACSDiva v8.0.1 and analyzed by FlowJo software (10.4).

### Lactic dehydrogenase (LDH) release

The quantitative assessment of cellular toxicity was conducted by means of a LDH release assay (Promega). Briefly, aliquots (50 μL) of the cell culture supernatants were transferred to a 96-well plate and then incubated with an equal volume of LDH substrate mixture for a period of 30 min at room temperature and protected from light. The reaction was terminated by adding 50 μL of stop solution, and absorbance was measured at 490 nm using a microplate reader (Tecan). All measurements were performed in triplicate with parallel blank corrections.

### In vivo NK cytotoxicity assay

For the in vivo Yac-1 clearance assay, CFSE-labeled Yac-1 cells were intraperitoneally injected into WT mice and KO mice. The mice were euthanized 24 h later, and cells in the peritoneal cavity were collected by repeatedly washing with PBS containing 2 mM EDTA. Then, surviving Yac-1 cells were identified by flow cytometry.

### RT-qPCR

Total RNA of cells was extracted using TRIzol (Invitrogen) methods, cDNAs were synthesized using M-MLV Reverse Transcriptase Kit (Invitrogen). The mRNA levels were tested by RT-qPCR using SYBR Green Real-time PCR Master Mix (Vazyme). The complete list of primers used is provided in Table S2.

### Western blot analysis

Protein was separated on a 10% gel by SDS-PAGE and subsequently electrotransferred to nitrocellulose. The membrane was blocked with 5% dry milk in TBST and probed with primary antibody. After incubation with horseradish peroxidase-conjugated secondary Ab, specific protein bands were detected by chemiluminescence.

### Single-cell RNA-seq analysis

Published single-cell RNA-seq datasets were analyzed by integrating the single-cell RNA-seq data visualization and analyzation (scDVA) web server (http://pan-nk.cancer-pku.cn/). This dataset included pan-cancer single-cell RNA expression of tumor-infiltrating NK cells [8]. To explore the relationship between EHMT2 and NK cell effector genes, an embedding plot, a significance plot and a heatmap plot were used.

### RNA-seq analysis

Raw reads were processed with fastp (v0.23.4) [25] and aligned to GRCm38/mm10 (mouse) or GRCh38/hg38 (human) using STAR (v2.7.11b) [26], allowing 2 mismatches. HTSeq (v2.0.3) [27] generated raw counts. DESeq2 [28] identified DEGs with |log2FC| > 0.5 and BH-adjusted *p* < 0.01.

### Gene expression microarray analysis

Data from GSE22919 (GPL570) were analyzed [29]. Probes were converted to gene symbols; multi-probe genes were averaged. Limma [30] identified DEGs with |log2FC| > 1 and *p* < 0.05.

### CUT&Tag analysis

CUT&Tag was performed on 1.6 × 10^5^ sorted NK cells using anti-H3K9me2. Libraries were sequenced, processed with fastp [25], and aligned to GRCm38/mm10 via Bowtie2 [31]. Bedtools [32] generated coverage files. Peaks were called with SEACR [33] (FDR < 0.01), annotated by HOMER [34], and visualized with DeepTools [35] and IGV [36]. MAnorm [37] identified differential peaks (|log2FC| > 0.3, BH-adjusted *p* < 0.05).

### Functional enrichment analysis

ClusterProfiler [38] identified enriched KEGG/GO-BP terms (BH-adjusted *p* < 0.05). GSEA assessed pathway activity (BH-adjusted *p* < 0.05; NES). Enrichplot visualized results.

### Statistical analysis

GraphPad Prism 10.0 software was used for data analysis. Normality of the data distribution was assessed using the Shapiro-Wilk test, and homogeneity of variances was evaluated by the *F*-test (for two-group comparisons) or Brown-Forsythe test (for multiple-group comparisons). For comparisons between two groups, a two-tailed unpaired Student’s *t*-test was performed. For non-normally distributed data, the Mann-Whitney U test was used. For multiple group comparisons (*n* ≥ 3), one-way analysis of variance (ANOVA) was conducted, followed by Tukey’s multiple comparisons test. Data obtained in experiments involving multiple donors were analyzed with a two-tailed paired Student’s *t*-test. For tumor growth curve analysis over time, statistical significance was evaluated using two-way ANOVA with Sidak’s post hoc test. Survival curves were compared using the log-rank test. The statistical significance is indicated for each experiment (*p < 0.05; **p < 0.01; ***p < 0.001; ****p < 0.0001). If not stated otherwise, all results are expressed as mean ± SEM. No statistical methods were used to pre-determine sample sizes; however, they are consistent with standard practices in the field. No animals or data points were excluded from the analyzes. Sample size (*n*) for each experiment is stated in the related figure legend.

### Ethics

The clinical study was approved by the Ethics Committee of Tianjin Medical University General Hospital (IRB2024-YX-563-01). Written informed consent was obtained from all participants prior to enrollment. All methods involving human participants were performed in accordance with the relevant guidelines and regulations. All animal experiments were approved by the Animal Ethics Committee of Capital Medical University (AEEI-2024-062). All animal procedures were performed in accordance with the relevant guidelines and regulations.

## Supplementary information

Supplementary files

Uncropped blot

## Data Availability

The data generated in this study are publicly available in GSE325753 and GSE325754 in the GEO database.
