# Supplementary material for: Targeting EHMT2 enhances NK cell maturation and antitumor activity
Source: Cell Death Dis. 2026 Jul 24;17(1):820. doi: 10.1038/s41419-026-09124-y (PMC13594311; doi:10.1038/s41419-026-09124-y)

Fig S3C

Ehmt2 Histone H3 H3K9me2

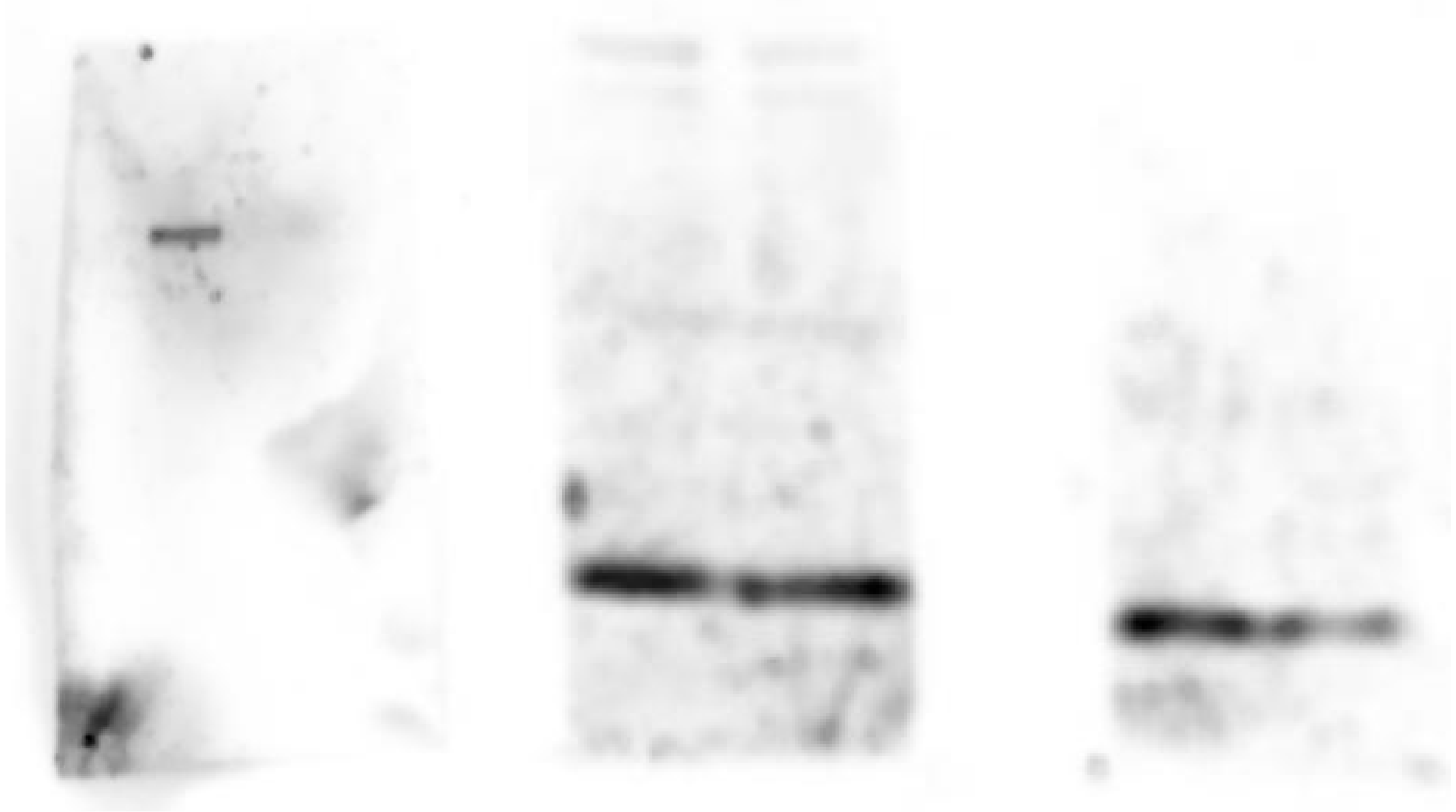

Fig S5A

Histone H3

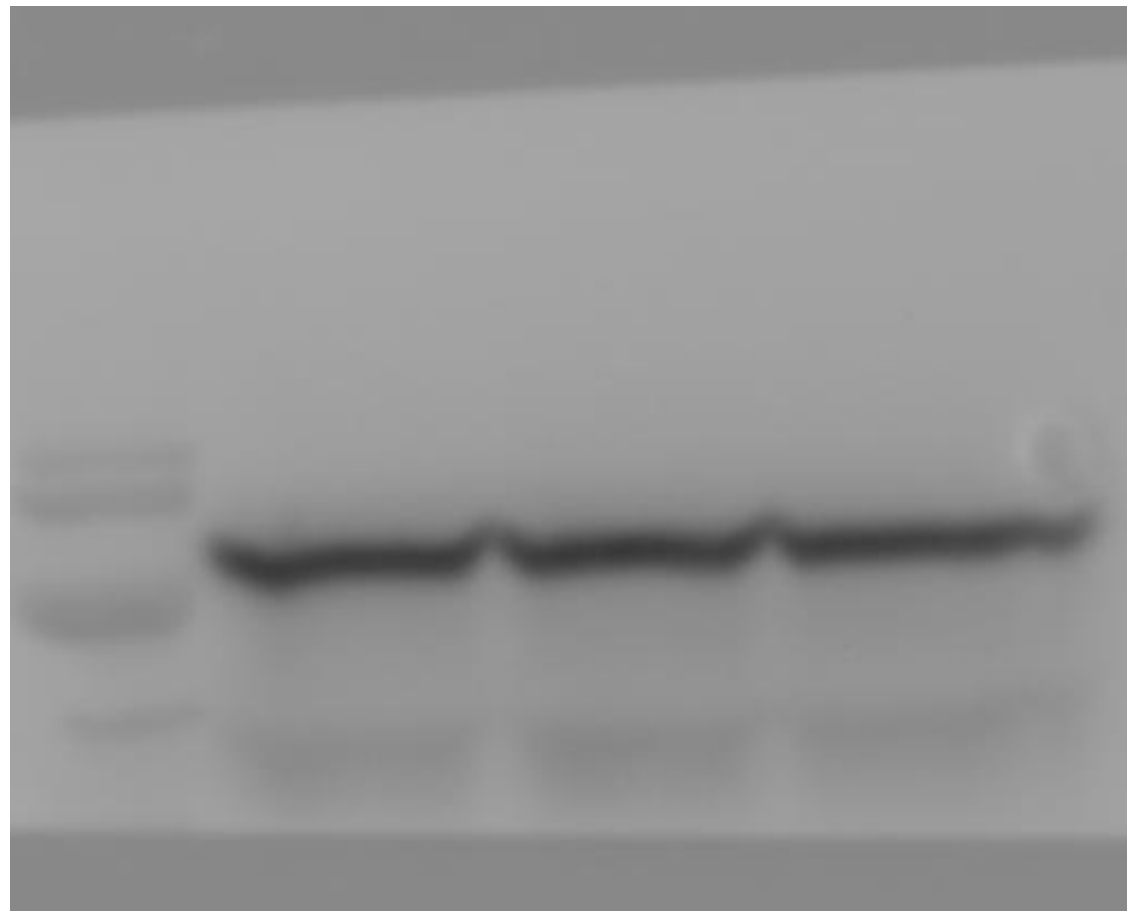

H3K9me2

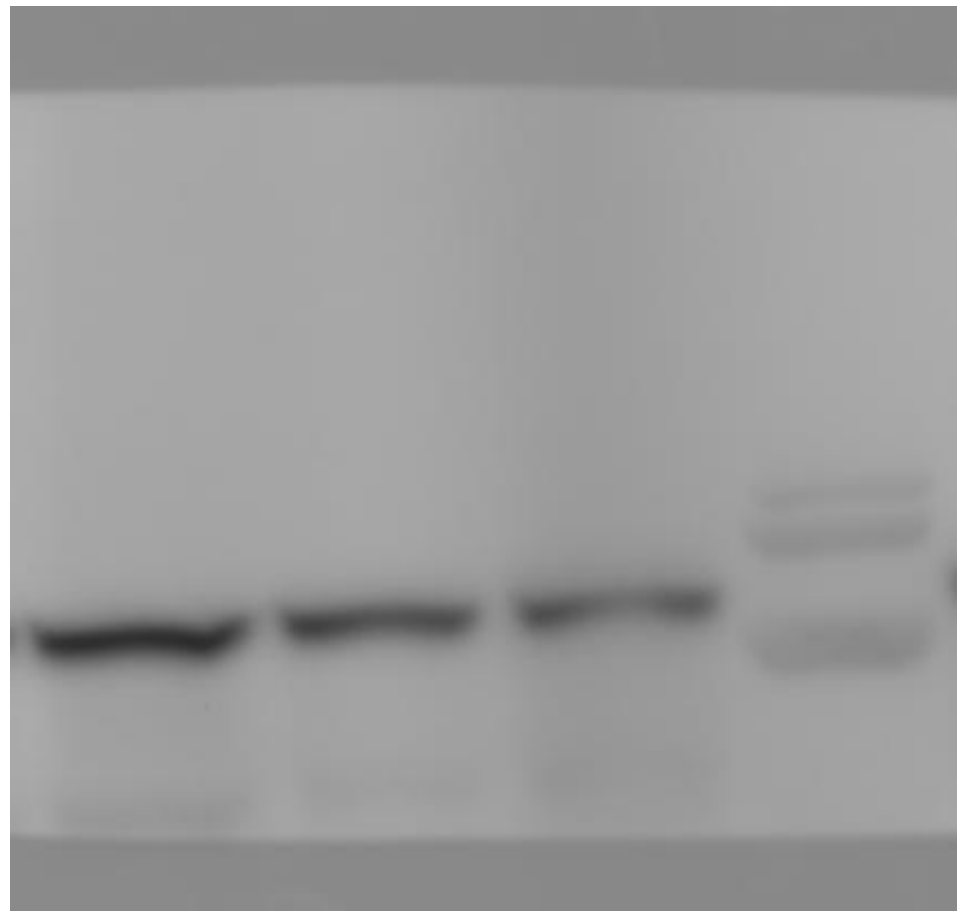

Fig S5C

Histone H3

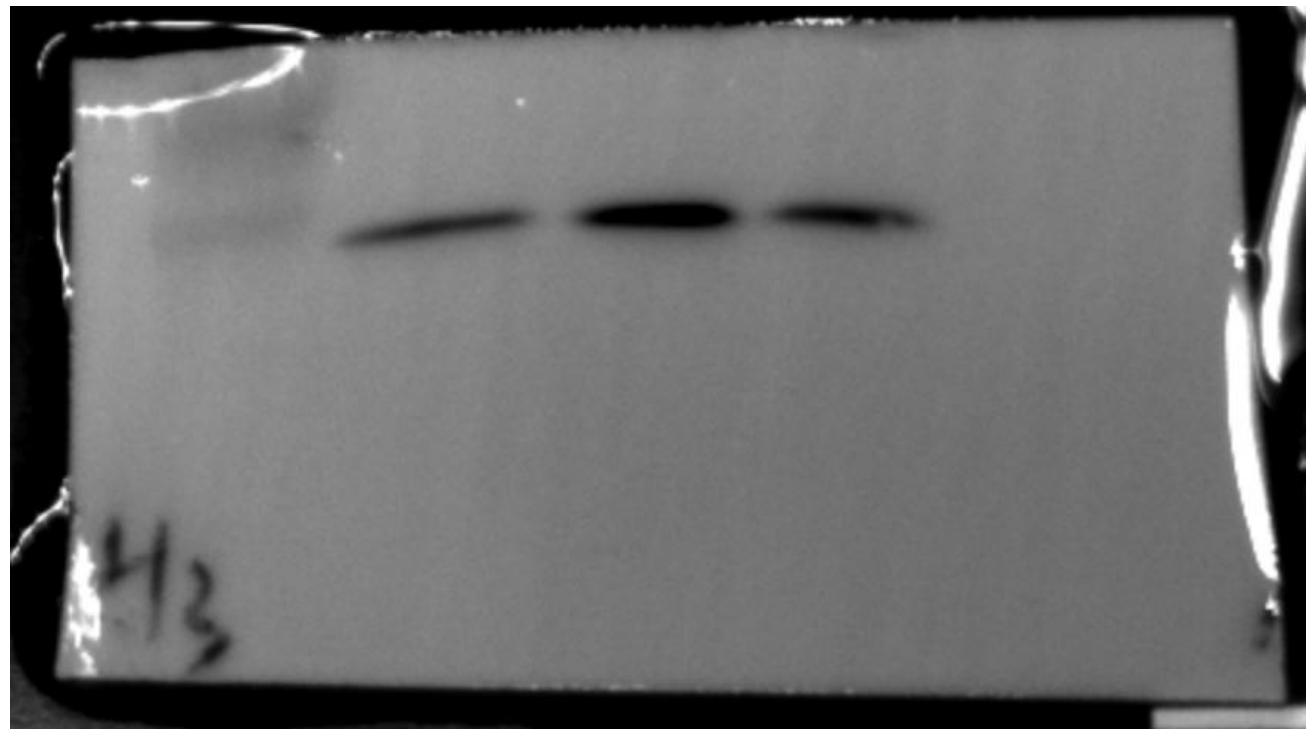

H3K9me2

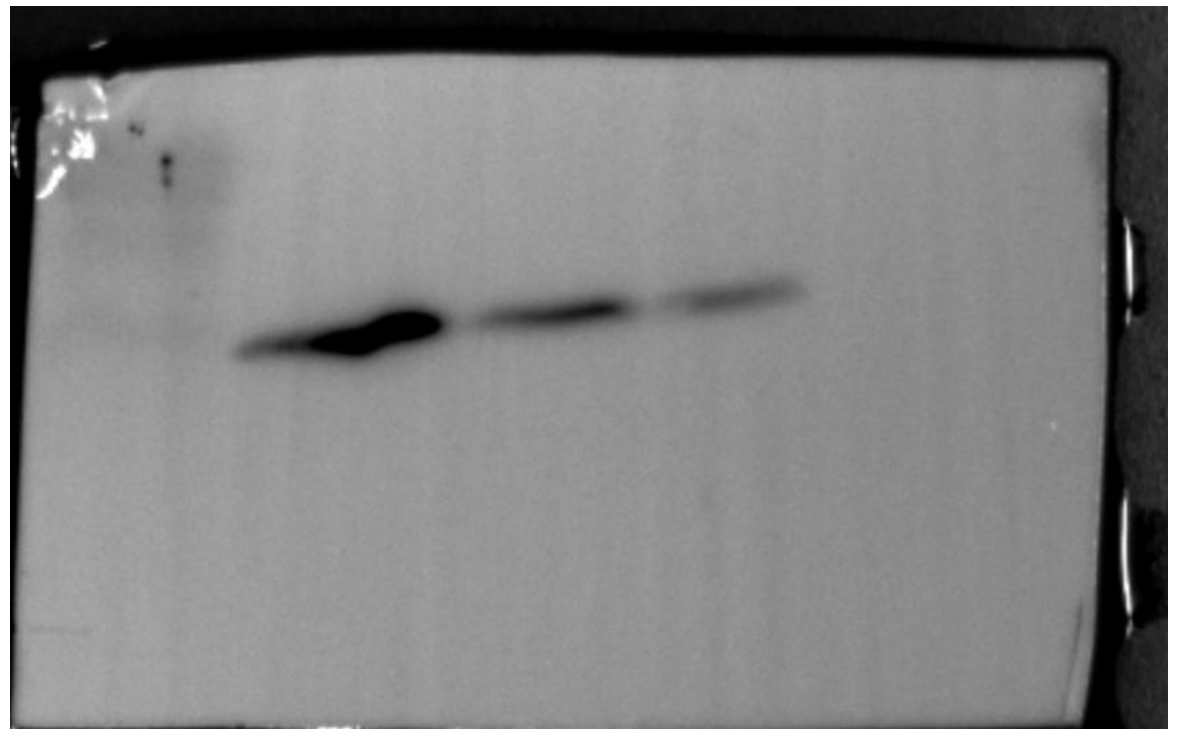

Fig S7A

β-action

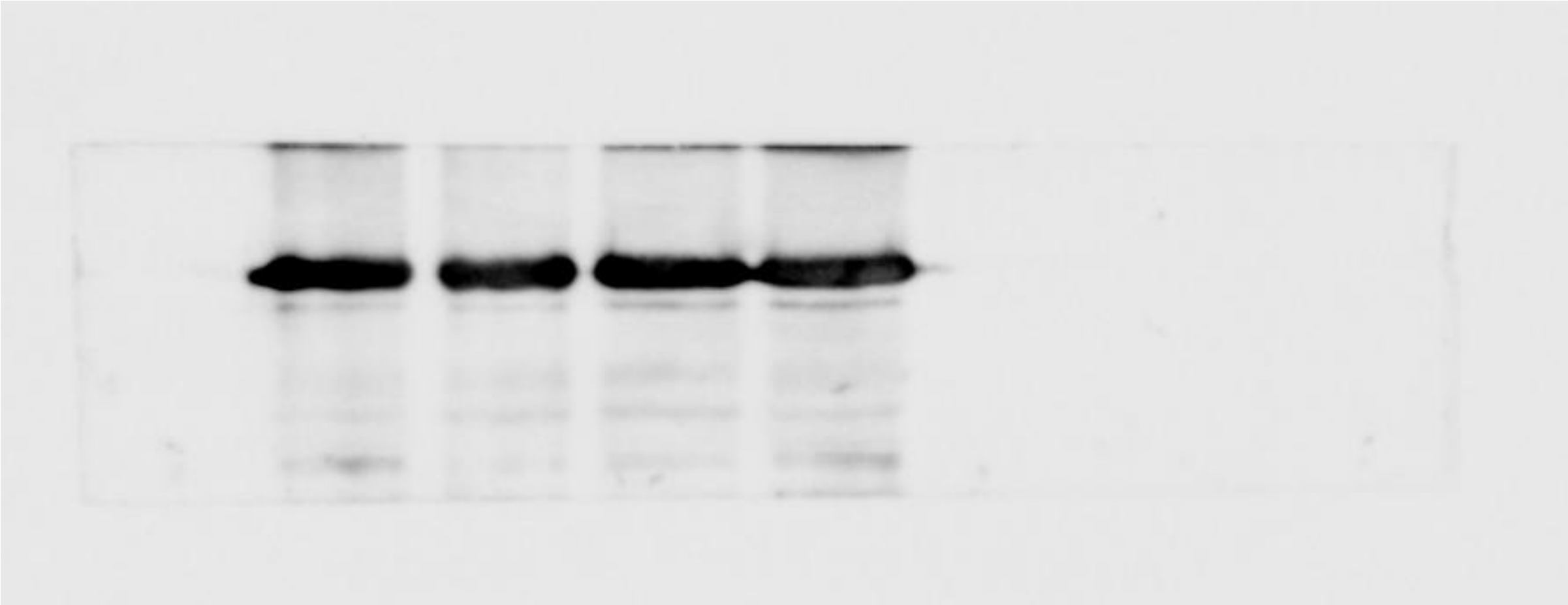

H3K9me2

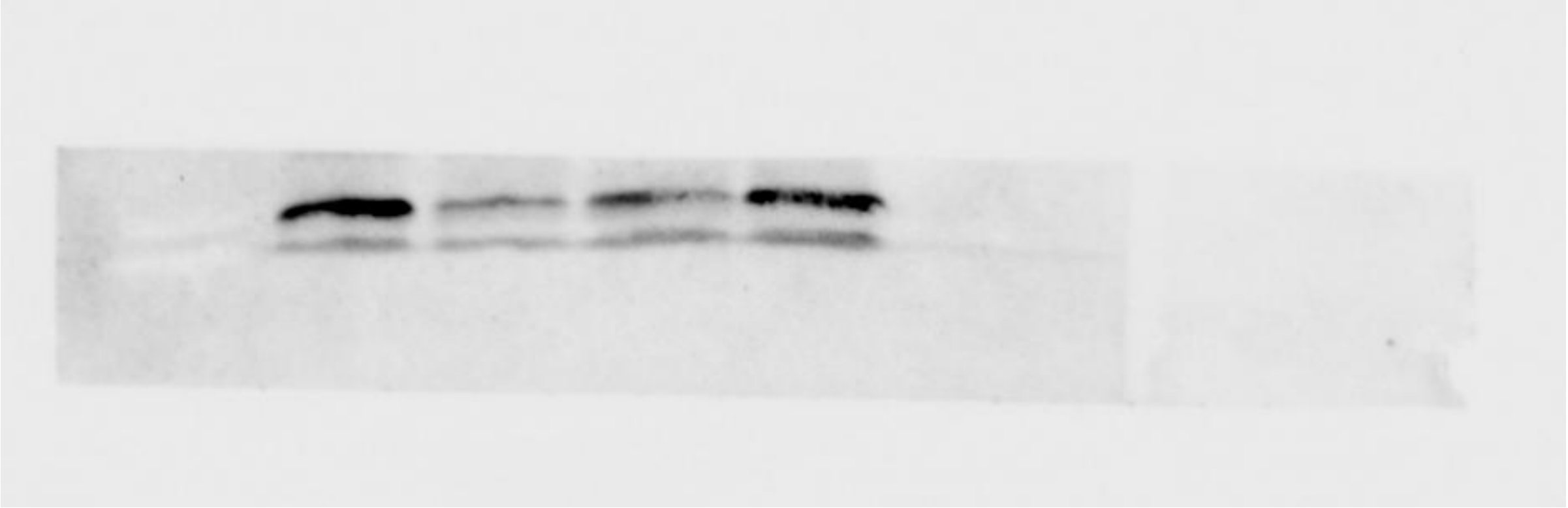

Fig S7F

β-action

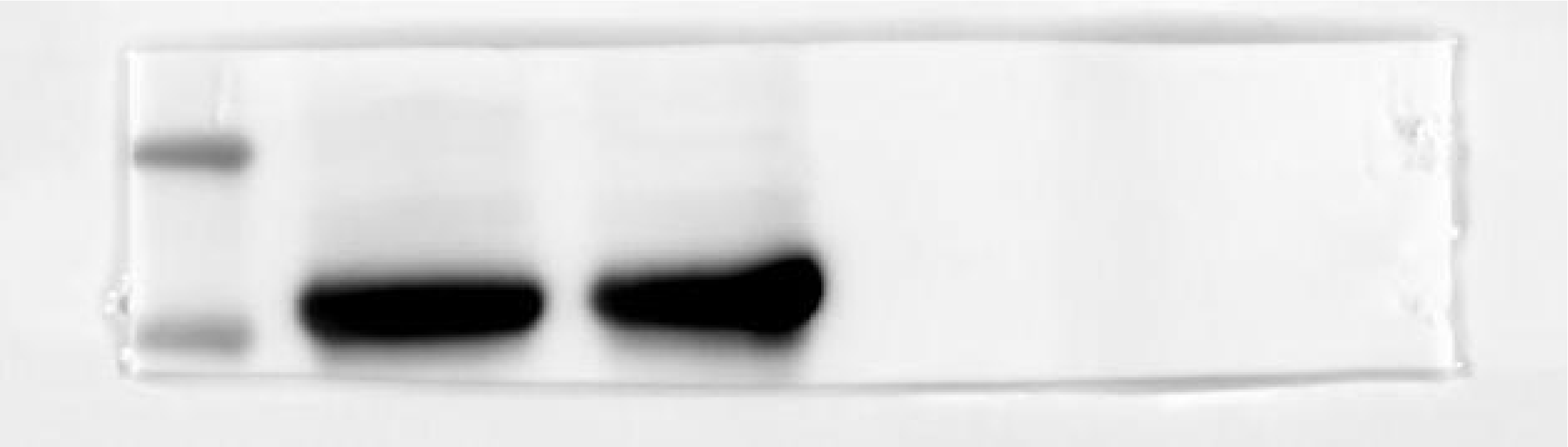

H3K9me2

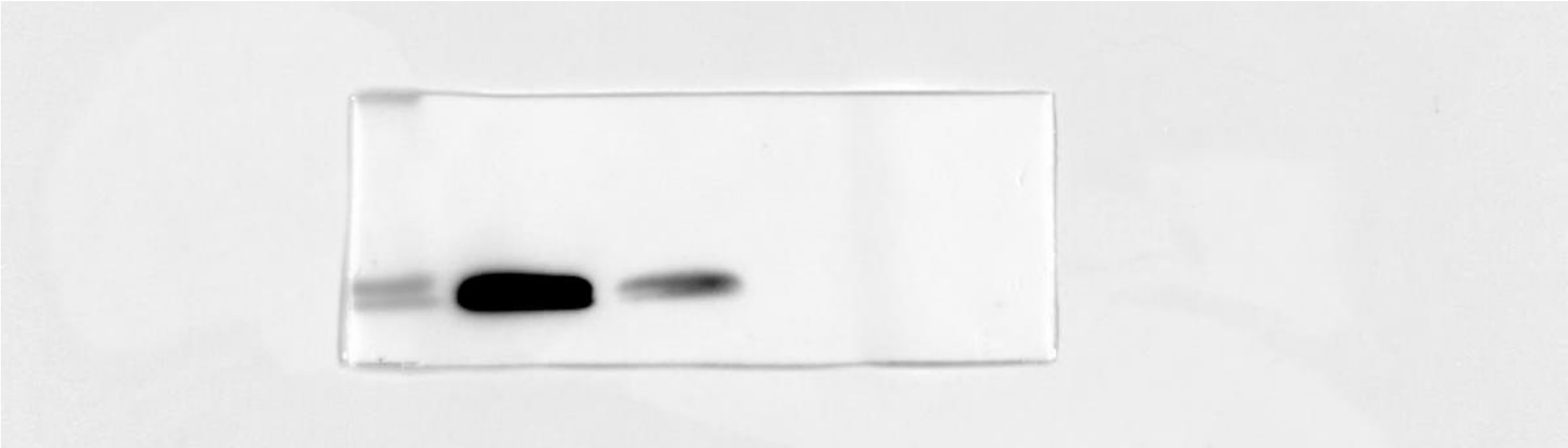

Fig S7L

β-action

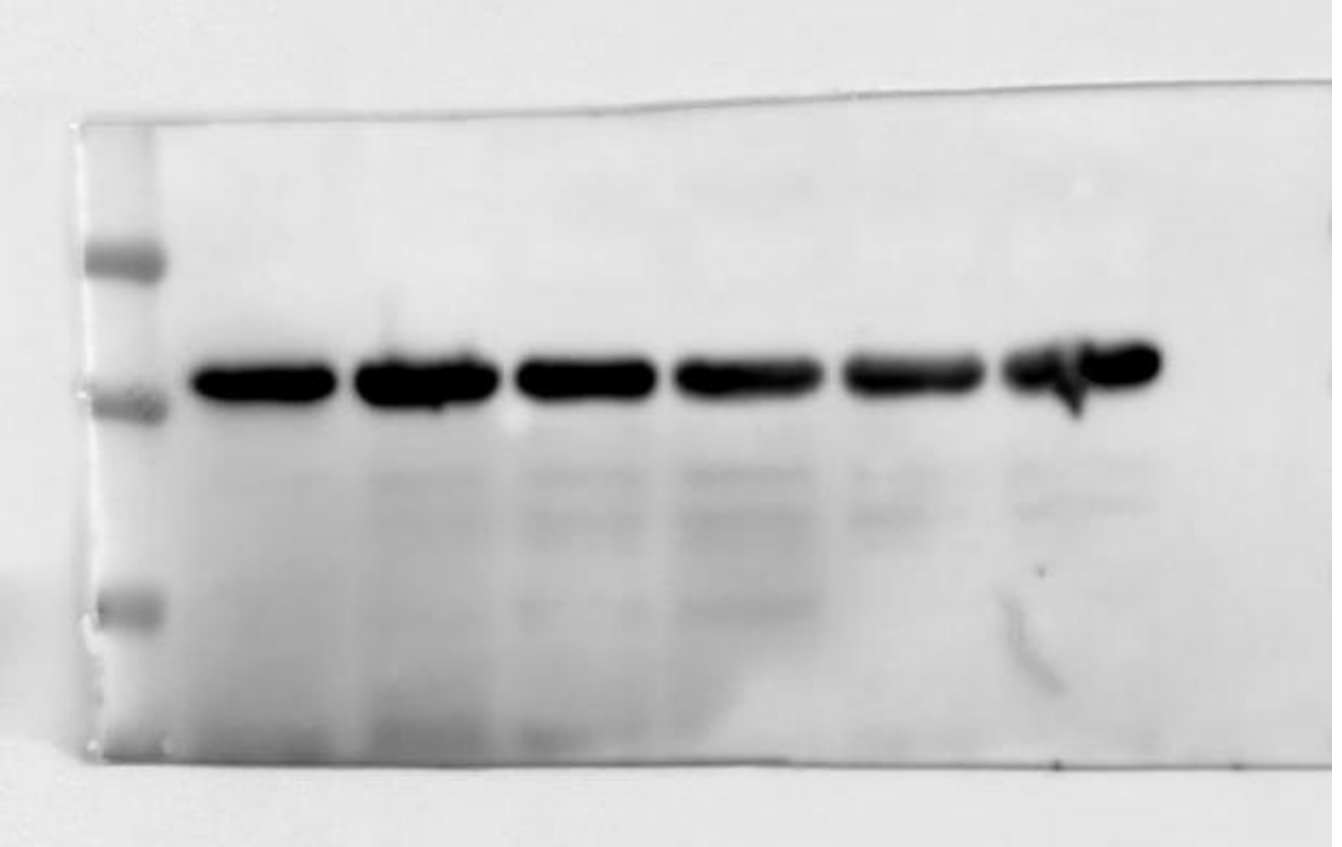

H3K9me2

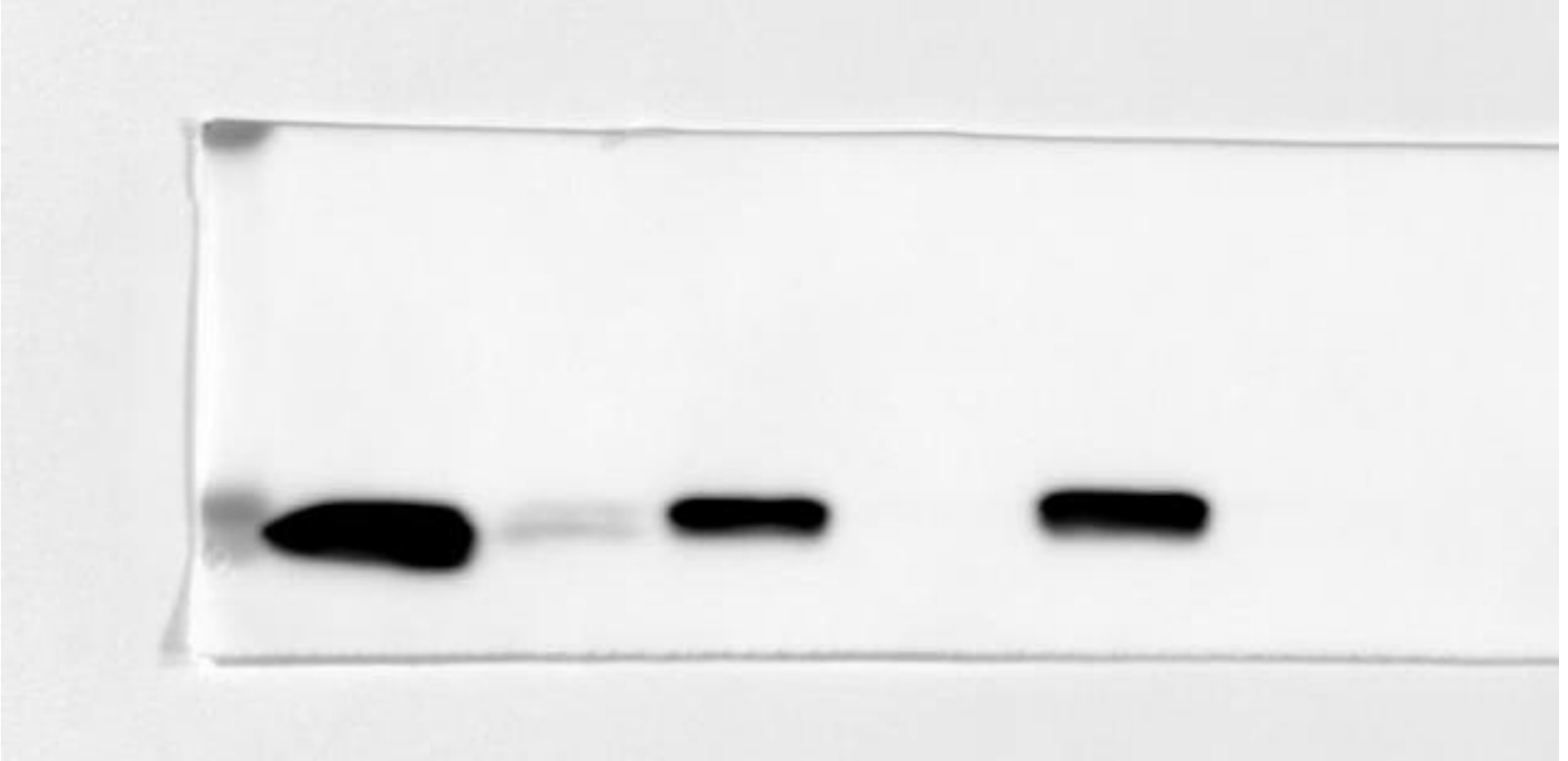

Supplement: Supplementary file 1 [file 41419_2026_9124_MOESM2_ESM.pdf]
