# Supplementary material for: Targeting EHMT2 enhances NK cell maturation and antitumor activity
Source: Cell Death Dis. 2026 Jul 24;17(1):820. doi: 10.1038/s41419-026-09124-y (PMC13594311; doi:10.1038/s41419-026-09124-y)
Supplement: Supplementary file 2 [file 41419_2026_9124_MOESM1_ESM.docx]

**Supplementary Files**

**Targeting EHMT2 Enhances NK Cell Maturation and Antitumor Activity**

Minghang Yu^1,2,3,9^, Xinhua Liu^4,9^, Wenxiu Zhang^1,9^, Xuefeng Huang^1^, Yue Wu^1^, Xinying Wang^4^, Yuanyuan Chen^5^, Ning Ding^6,*^, Zilong Zhao^7,*^, Xi Wang^1,2,3,*^, Yang Li^8,*^

^1^National Key Laboratory of Intelligent Tracking and Forecasting for Infectious Diseases, Beijing Key Laboratory of Viral Infectious Diseases, Institute of Infectious Diseases, National Center for Infectious Diseases, Beijing Ditan Hospital, Capital Medical University, Beijing, China.

^2^Beijing Institute of Infectious Diseases, Beijing, China.

^3^Department of Oncology, Capital Medical University, Beijing, China.

^4^Department of Biochemistry and Molecular Biology, School of Basic Medicine Sciences, Hangzhou Normal University, Hangzhou, Zhejiang, China.

^5^Department of Gynecology and Obstetrics, Tianjin Key Laboratory of Female Reproductive Health and Eugenics, Tianjin Medical University General Hospital, Tianjin, China.

^6^Key Laboratory of Carcinogenesis and Translational Research, Laboratory of Lymphoma Translational Research, Peking University Cancer Hospital & Institute, Beijing, China.

^7^Department of Neurosurgery, Tianjin Institute of Neurology, Tianjin Medical University General Hospital, Tianjin, China.

^8^Precision Medicine Center, Tianjin Medical University General Hospital, Tianjin, China.

^9^These authors contributed equally

^*^Correspondence: ningding@bjmu.edu.cn (N.D.), zilongzhaotianjin@hotmail.com (Z.Z.), xiwang@ccmu.edu.cn (X.W.), liyang_tmugh@tmu.edu.cn (Y.L.).

**Figure S1. Cytokine stimulation remodels the human NK cell transcriptome**

(A) PCA analysis of human NK cells from two donors, grouped as stimulated (with IL-2, IL-12, and IL-18 for 24 hours) and unstimulated controls. (B) Volcano plot showing DEGs in cytokine-stimulated human NK cells compared to unstimulated NK cells. Significant alterations in the expression levels of 4 775 genes (including 2 704 downregulated genes and 2 071 upregulated genes) were observed following cytokine stimulation (|log_2_FoldChange| > 1 and *p* < 0.05). (C-D) GO (C) and KEGG (D) enrichment analysis of DEGs in human NK cells from stimulated versus unstimulated NK cells. Dot sizes represent the counts of DEGs in a pathway, and dot colors from red to blue indicate adjusted *p* values from low to high. (E) Heatmap of methyltransferase and demethyltransferase expression profiles between cytokine-stimulated human NK cells and unstimulated NK cells. (F) EHMT2 mRNA levels in human NK cells were measured by RT-qPCR (*n* = 3 donors). The data are shown as the mean ± SEM. ***p* < 0.01.

**Figure S2. Expression profiles of key cytotoxic molecules GZMB and PRF1**

(A-B) Heatmaps depict expression variations of GZMB (A) and PRF1 (B) in tumor, non-tumor, blood, and other tissue categories. Each box represents pairwise comparison test results between the group indicated in the row header and the column header. Numerical values within boxes denote log_2_FoldChange, while color saturation corresponds to differential significance (darker hues indicate higher significance; default significance threshold by 0.05 and only the significant comparisons are marked). Red/blue coloring exclusively reflects positive/negative log_2_FoldChange values. Pie charts display the percentage of cells expressing each gene within respective groups.

**Figure S3. Generation and validation of Ehmt2 KO mice**

(A) Schematic diagram showing the targeting strategy for generating Ehmt2^flox/flox^ mice. (B) Genotyping PCR analysis of genomic DNA from WT and Ehmt2 KO mice. (C) Western blot analysis of Ehmt2 and H3K9me2 protein levels in splenic NK cells of WT and Ehmt2 KO mice, with histone H3 as a loading control. (D-E) Ifng (D) and Prf1 (E) mRNA expression detected by RT-qPCR in WT and KO NK cells. The data are shown as the mean ± SEM. The data are representative of at least two independent experiments. **p <* 0.05.

**Figure S4. Ehmt2 deficiency alters the expression of activation-related molecules in NK cells**

(A-E) Heatmap of activating receptors (A), inhibitory receptors (B), transcription factors (C), effector genes (D) and cytokine receptors (E) between WT NK cells and KO NK cells. (F)Venn diagram showing the overlap between 3 125 genes associated with 12 747 downregulated peaks (|log_2_FoldChange| > 0.3 and adjusted *p* value < 0.05) identified by CUT&Tag analysis and 1 031 upregulated genes (|log_2_FoldChange| > 0.3 and adjusted *p* value < 0.01) detected by RNA-seq. A total of 199 genes were shared between the two datasets.

**Figure S5. Pharmacological inhibition of EHMT2 enhances NK92MI cell effector functions**

(A) Western blot analysis of H3K9me2 and Histone H3 in primary NK cells treated with DMSO or BIX-01294. (B) IFNG mRNA levels in primary NK cells treated with DMSO or BIX-01294 were analyzed by RT-qPCR. The cells were co-stimulated with PMA and ionomycin. (C) Western blot analysis of H3K9me2 and total H3 in NK92MI cells treated with DMSO or BIX-01294. (D) The mRNA levels of IFNG in NK92MI cells in the EHMT2 inhibitor or DMSO groups were analyzed by RT-qPCR. The cells were co-stimulated with PMA and ionomycin. (E-F) Flow cytometry plots (left) and scatter plots (right) show intracellular staining of IFN-γ (E) and Granzyme B (F) by unstimulated NK92MI cells in the BIX-01294 or DMSO groups. (G-I) Flow cytometry plots show intracellular staining of IFN-γ production by NK92MI cells in the BIX-01294 or DMSO groups. The cells were co-stimulated with PMA and ionomycin (G), IL-2 + IL-12 + IL-18 (H) or K562 cells (I) in the presence of GolgiStop. (J) HepG2 cells specifically lysed by NK92MI cells from BIX-01294 or DMSO groups at the indicated E:T ratios (n = 3 per group; at least two independent experiments). NK92MI cells were treated for 5 days with 0.25 μM BIX-01294 or DMSO. The data are shown as the mean ± SEM. **p* < 0.05; ***p* < 0.01; ****p* < 0.001; *****p* < 0.0001.

**Figure S6. EHMT2 inhibition drives a transcriptomic cascade enriching for NK cell activation-related gene sets**

(A) PCA of gene expression in NK92MI cells from the BIX-01294 and DMSO groups. (B) Volcano plot showing DEGs in NK92MI cells between the BIX-01294 and DMSO groups. (C) KEGG pathway analysis of genes that are enriched in NK cells between the BIX-01294 and DMSO groups. (D) GO enrichment analysis of DEGs in NK cells from the BIX-01294 and DMSO groups. Dot sizes represent the counts of DEGs in a pathway, and dot colors from red to blue indicate adjusted *p* values from low to high. (E-F) GSEA illustrating enrichment of DNA binding transcription activator activity (E) and positive regulation of natural killer cell activation (F) gene sets in the BIX-01294 and DMSO groups. (G-J) Heatmap of effector molecule (G), cytokine receptor (H), activating receptor (I) and inhibitory receptor (J) in NK92MI cells in the BIX-01294 and DMSO groups.

**Figure S7. Validation of the specificity of EHMT2 inhibition on NK cell functions**

(A) Western blot analysis of H3K9me2 levels in WT+DMSO, WT+BIX, and KO+BIX groups. β-actin was used as a loading control. (B-D) Representative flow cytometry histograms (left) and statistical analysis of MFI (right) for IFN-γ (B), Perforin (C), and Granzyme B (D) in the indicated groups (*n* = 6). (E) Yac-1 cells specifically lysed by NK cells from WT+DMSO, WT+BIX, and KO+BIX groups at the indicated E:T ratios (*n* = 3 per group; at least 3 independent experiments). Statistical analysis at a 10:1 E:T ratio was shown. (F) Western blot analysis of H3K9me2 levels in DMSO and UNC0642 groups. β-actin was used as a loading control. (G-I) Representative flow cytometry histograms (left) and paired scatter plots (right) show intracellular staining of IFN-γ (G), Perforin (H), and Granzyme B (I) by NK cells in the UNC0642 or DMSO groups (*n* = 5 donors). (J) Cell-mediated cytotoxicity of NK cells in the UNC0642 or DMSO groups against HepG2 cell lines at indicated E:T ratios. Graphs show percent-specific lysis for NK cells from one representative donor. (K) Relative change in specific lysis was calculated for NK cells in the UNC0642 or DMSO groups at a 1:1 E:T ratio (*n* = 5 donors). (L) Western blot analysis of H3K9me2 levels in NK92MI cells transduced with sgCtrl or sgEHMT2. β-actin was used as a loading control. (M-O) Representative flow cytometry histograms (left) and scatter plots (right) show intracellular staining of IFN-γ (M), TNF-α (N), and Perforin (O) by NK cells in the sgCtrl+DMSO, sgCtrl+BIX and sgEHMT2+DMSO groups (*n* = 4). **p* < 0.05; ***p* < 0.01; ****p* < 0.001; *****p* < 0.0001.

Table S1: Antibody list

| Antibody | Fluorochrome | Clone | Company | Cat# |
| --- | --- | --- | --- | --- |
| anti-mouse NK1.1 | APC | PK136 | BD Biosciences | 561117 |
| anti-mouse CD27 | PE | LG.3A10 | BD Biosciences | 561785 |
| anti-mouse NKp46 | FITC/PE | 29A1.4 | BD Biosciences | 560756/560757 |
| anti-mouse CD3ε | BV421/FITC | 17A2/145-2C11 | BioLegend | 100214/100306 |
| anti-mouse CD49b | PE | DX5 | BioLegend | 108908 |
| anti-mouse CD122 | PE | TM-β1 | eBioscience | 12-1222-83 |
| anti-mouse CD11b | AF700 | M1/70 | BD Biosciences | 557960 |
| anti-mouse KLRG1 | PE | 2F1 | eBioscience | 12-5893-82 |
| anti-mouse Granzyme B | PE | QA16A02 | BioLegend | 372208 |
| anti-mouse Perforin | APC | S16009A | BioLegend | 154304 |
| anti-mouse IFN-γ | APC | XMG1.2 | BioLegend | 505810 |
| anti-human CD56 | BV605 | NCAM16.2 | BD Biosciences | 562780 |
| anti-human NKp46 | APC | 9E2 | BioLegend | 331918 |
| anti-human NKG2D | PE | 1D11 | BioLegend | 320806 |
| anti-human CD3 | PE-dazzle594 | UCHT1 | BioLegend | 300450 |
| anti-human IFN-γ | eF450 | 4S.B3 | eBioscience | 48-7319-42 |
| anti-human Granzyme B | AF647 | GB11 | BD Biosciences | 561999 |
| anti-human TNF-α | PE-Cy7 | MAb11 | eBioscience | 25-7349-82 |
| anti-human-Perforin | BV421 | δG9 | BD Biosciences | 563393 |
| [anti-H3K9me2](https://www.affbiotech.com/goods-5739-DF6937-Di_Methyl_Histone_H3_Lys9_H3K9me2_Antibody.html) | - | - | [Affinity Biosciences](https://www.affbiotech.com/)/Abcam | [DF6937](https://www.affbiotech.com/goods-5739-DF6937-Di_Methyl_Histone_H3_Lys9_H3K9me2_Antibody.html)/ab176882 |
| anti-EHMT2 | - | - | Abcam | ab185050 |
| anti-Histone H3 | - | - | Abcam | ab1791 |
| anti-beta Actin | - | - | [Affinity Biosciences](https://www.affbiotech.com/)/Abcam | AF7018/ab6276 |

Table S2: Primer list for RT-qPCR

| Primer | Sequence (5’-3’) |
| --- | --- |
| h-EHMT2-F | CTGTCAGAGGAGTTAGGTTCTGC |
| h-EHMT2-R | CTTGCTGTCGGAGTCCACG |
| h-IFN-γ-F | TCGGTAACTGACTTGAATGTCCA |
| h-IFN-γ-R | TCGCTTCCCTGTTTTAGCTGC |
| m-Gzmb-F | TCTCGACCCTACATGGCCTTA |
| m-Gzmb-R | TCCTGTTCTTTGATGTTGTGGG |
| m-Tbx21-F | AACCGCTTATATGTCCACCCA |
| m-Tbx21-R | CTTGTTGTTGGTGAGCTTTAGC |
| m-Il2rb-F | CCTTTGACAACCTTCGCCTG |
| m-Il2rb-R | CCCGAGGATCAGGTTGCAG |
| m-Id2-F | TCCGGTGAGGTCCGTTAGG |
| m-Id2-R | CAGACTCATCGGGTCGTCC |
| m-Prf1-F | CAAGGTAGCCAATTTTGCAGC |
| m-Prf1-R | GTACATGCGACACTCTACTGTG |
| m-Ifng-F | ATGAACGCTACACACTGCATC |
| m-Ifng-R | CCATCCTTTTCAGTTCCTC |
